# Vulnerability of the British swine industry to classical swine fever

**DOI:** 10.1038/srep42992

**Published:** 2017-02-22

**Authors:** Thibaud Porphyre, Carla Correia-Gomes, Margo E. Chase-Topping, Kokouvi Gamado, Harriet K. Auty, Ian Hutchinson, Aaron Reeves, George J. Gunn, Mark E. J. Woolhouse

**Affiliations:** 1 Epidemiology Research Group, Centre for Immunity, Infection and Evolution, University of Edinburgh, Edinburgh, Scotland, UK; 2 Epidemiology Research Unit, Future Farming Systems, Scotland’s Rural College, Inverness, Scotland, UK; 3 Biomathematics & Statistics Scotland, Edinburgh, Scotland, UK

## Abstract

Classical swine fever (CSF) is a notifiable, highly contagious viral disease of swine which results in severe welfare and economic consequences in affected countries. To improve preparedness, it is critical to have some understanding of how CSF would spread should it be introduced. Based on the data recorded during the 2000 epidemic of CSF in Great Britain (GB), a spatially explicit, premises-based model was developed to explore the risk of CSF spread in GB. We found that large outbreaks of CSF would be rare and generated from a limited number of areas in GB. Despite the consistently low vulnerability of the British swine industry to large CSF outbreaks, we identified concerns with respect to the role played by the non-commercial sector of the industry. The model further revealed how various epidemiological features may influence the spread of CSF in GB, highlighting the importance of between-farm biosecurity in preventing widespread dissemination of the virus. Knowledge of factors affecting the risk of spread are key components for surveillance planning and resource allocation, and this work provides a valuable stepping stone in guiding policy on CSF surveillance and control in GB.

Classical swine fever (CSF) is a notifiable, highly contagious viral disease of swine, both domestic and wild, which results in severe animal welfare and economic consequences in affected countries^1^,^2^. Because of the potential for severe socio-economical costs associated with CSF^3^, efforts have been carried out to control and eradicate CSF in the European Union (EU) with a common strategy implemented by EU legislation (Council Directive 2001/89/EC). During the last decade, however, CSF has circulated within wild boar population in different European countries, including several EU Member States, and the Russian Federation, posing an ongoing threat to swine industries in the rest of Europe^1^,^4^. Although there are regulations in place to reduce the risk of CSF spread within Europe (for example regarding the import of live animals and the feeding of food waste), the risk of CSF incursions still remains. Most introductions of CSF into EU member states were related to the purchase of animals, feeding contaminated pork products to pigs, and direct or indirect contact with wild boars^5^. In Great Britain (GB), the last outbreak of CSF that occurred in GB in 2000 was found caused by legal and illegal imports of contaminated pork products^6^.

Once the detection of CSF virus in a farm occurs, strict measures are implemented to control disease spread, including depopulating infected premises (IPs), tracing high risk premises, and strict movement restrictions for farms in the vicinity of IPs, in line with domestic and European level legislation. Depopulation of IPs is aimed at limiting disease spread by removing sources of infection, whilst movement restrictions are designed to prevent the transmission of CSF virus to naïve animals through direct contact with infected animals or via contaminated fomites. The impact of various factors on the effectiveness of these mitigation strategies in controlling disease have been described for CSF in other countries, or for other notifiable disease that spread in similar ways. In particular: (*i*) the location and timing of the incursion, which relate to farm density and the frequency of animal movements to or from the area^7^,^8^,^9^; (*ii*) the strain of the virus^7^; (*iii*) the efficacy of surveillance systems for rapid detection of incursions^10^; and (*iv*) human behaviour and the degree of compliance with control measures such as movement restrictions^11^,^12^ have all been found to affect the effectiveness of control. However to date, little information exists on the impact these factors may have on the risk of CSF spread in the British swine industry. Such information is critical to optimise surveillance and control strategies and ensure that CSF epidemics are detected and eradicated quickly and cost-effectively.

Mathematical models that simulate the course of epidemics are regularly used to provide guidance for decision-making to control outbreaks of notifiable diseases. Parameterisation of these models can be challenging and informing policy based on unrepresentative parameters could have negative consequences for disease management. In particular, important determinants in between-farm spread of disease^13^,^14^, such as pig density in the high production regions, national average pig farm density and farming structure, vary between countries. If available, historical outbreak data from the country of interest provide the opportunity to develop country-specific data-derived parameters. In this study, we used data collected during the last epidemic of CSF in GB to inform a spatially explicit, premises-based model, and evaluated how CSF may spread from single incursion events in the British pig industry. Specifically, we investigated (1) how the risk of CSF spread and the size of generated epidemics vary with the time taken to detect an incursion as well as the geographical location and the producer type of the primary case, and (2) explore which other factors involved in the transmission and control of CSF would influence the risk of spread and the size of generated epidemics. These are particularly relevant to improving CSF preparedness in GB.

## Results

### Overall risk

**Figure 1:**
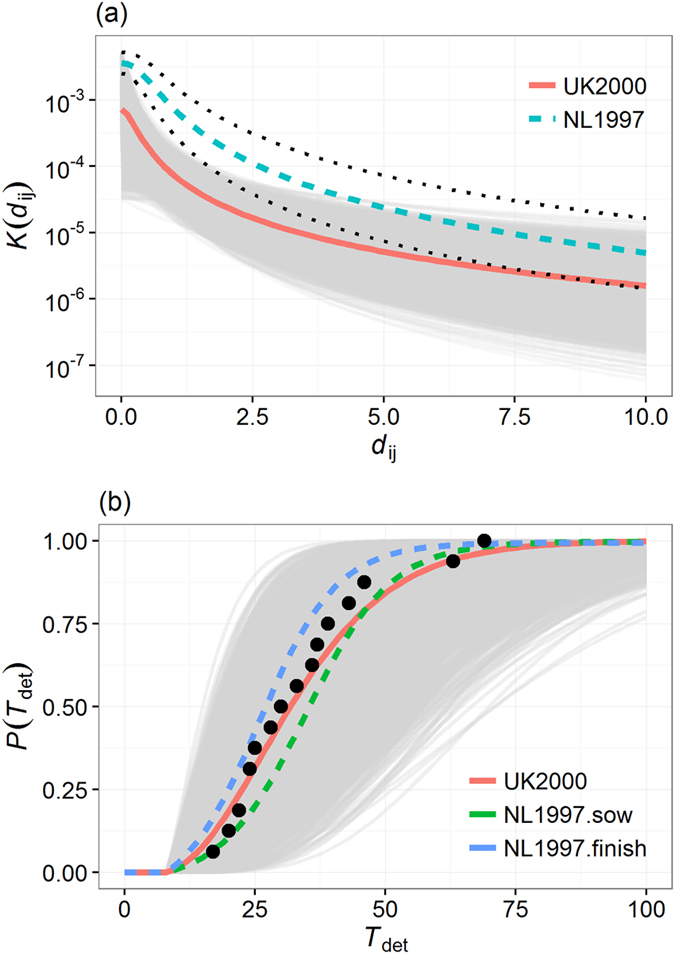
Fitted functions from the 2000 CSF epidemics in East Anglia, Great Britain. (**a**) Posterior estimate of the between-premises transmission function *K(d*_ij_), defined as the daily rate at which a given infected premises *j* may infect a susceptible premises *i* as a function of inter-premises distance *d*_ij_. (**b**) Cumulative probability function of the posterior estimate of the time of detection *T*_det_ for CSF-infected premises. Solid line is the fitted curve from the 2000 CSF epidemic in East Anglia. For comparison, equivalent curves fitted from the 1997 CSF epidemic in the Netherlands^14^,^15^ are also shown (dashed lines). Grey thin solid lines represent all posterior estimates of *K(d*_ij_) and *T*_det_ that are within the 95% credible interval around the median. Dotted lines in panel (a) show the confidence bounds of *K(d*_ij_) fitted from the 1997 CSF epidemic in the Netherlands. Note that the *y*-axis in panel (a) is log_10_-scaled. Solid dots in panel (b) represent the believed detection period informed through independent, field-based contact tracing.

The model developed in this study incorporates the transmission of disease from premises to premises through the movement of pigs and through local spread following the introduction of CSF in a single randomly selected farm in GB. The population consisted of all premises (i.e. farms and gathering places) present in 2012 and 2013. For simplicity, however, we considered incursions of CSF only occurring in a single, randomly chosen farm and within the period January 1^st^ 2012 to December 31^st^ 2012. The model also incorporates estimates of the time required to detect infected farms, which in turn drives the implementation of control activities. Functions informing the extent of the local spread (as defined by the “transmission kernel function” *K(d*_ij_), Fig. 1a) and the duration of the detection period during the active surveillance period (as defined by the “detection function” *T*_det_, Fig. 1b) were both fitted over the 16 cases reported during the 2000 CSF epidemic in East Anglia, GB. Here, the fitted functions *K(d*_ij_) and *T*_det_ were considered as national averages for CSF dispersal and detection for all infected farms, regardless of their production type, population size and within-farm prevalence. Figure 1 shows the shape of these two functions, their uncertainty and a comparison with those reported in the published literature^14^,^15^. Although the estimated shape of *T*_det_ from the UK is similar to those from the Netherlands, this was not the case for the local spread function *K(d*_ij_). The probability at which given infected premises may infect susceptible premises estimated from the UK outbreak was significantly (P < 0.05) lower for distances between 0.08 km to 4.35 km than that estimated from the Dutch outbreak in 1997/1998 (Fig. 1a). Notably, Fig. 1a highlights that the rate of spread from infected premises beyond 2 km was negligible (*K*(2 km) = 0.024. 95% Cr. I. = 0.004–0.083 per 1000 infectious premises per day).

**Figure 2:**
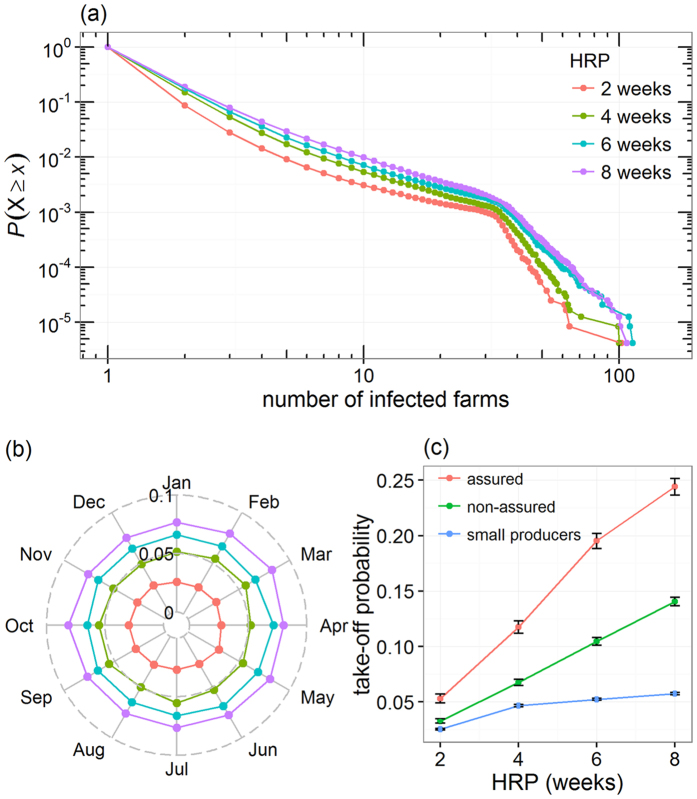
Temporal variation in the risk of CSF spread in Great Britain. (**a**) Decreasing cumulative probability function of the total number of infected farms involved in epidemics generated from a single incursion for various duration of the high risk period (HRP). (**b,c**) Changes in the overall probability of epidemic take-off generated from a single incursion as a function of (**b**) the month of the incursion and HRP duration or (**c**) the producer type of the farm in which incursion occurred. The HRP is defined as the length of time between the incursion and the date of the first detected infected premises. The probability of epidemic take-off is defined as the probability that a primary case generates epidemics which involves at least two other farms. Note that both axes in panel (a) are log_10_-scaled.

The “high risk period” (HRP) is the length of time between introduction and detection of a disease, during which no control measures have yet been implemented and disease continues to spread. In previous CSF outbreaks, the HRP has ranged from 2 to 9 weeks^6^,^16^ despite efforts to raise the awareness and vigilance of farmers and veterinary services. Once disease is detected, mitigation procedures are initiated within 24 hours of the first report and remain active until no infectious cases are present in the population. The descending cumulative probability distribution of the number of infected farms (including primary cases) involved in outbreaks generated by single incursion events and with a HRP lasting from 2 to 8 weeks is shown in Fig. 2a.

Over the 240,000 simulations carried out per HRP scenario, incursions would be restricted to the primary case in 91.3%, 84.9%, 82.5%, 81.3% of the time for a HRP of 2, 4, 6 and 8 weeks, respectively (Table 1 and Fig. 2a). Although all incursions would be detected if the HRP lasted no more than 2 weeks, only 15%, 8.5%, 8.0% of the epidemics would last long enough to be detected when the duration of the HRP is 4, 6 and 8 weeks, respectively. When detected, the median duration of the epidemic (defined as the length of time between detection of the disease to the depopulation of the last IP) was 1 day (95% range 1–30 days), 15 days (95% range 2–88 days), 20 days (95% range 1–105 days) and 21 days (95% range 1–109 days) when the HRP lasts 2, 4, 6 and 8 weeks, respectively (Table 1). In these situations, the median number of infected farms would be 1 (95% range 1–3), 2 (95% range 1–25), 3 (95% range 1–25) and 3 (95% range 1–30) farms, respectively. However, substantial disease spread may still occur regardless of the HRP duration. For a HRP of 2, 4, 6 and 8 weeks, as many as 102, 100, 113 and 107 farms were infected during at least one of the simulation runs, respectively (Table 1).

To investigate the likelihood that an epidemic may be generated from a single incursion event in GB, we assessed the probability of epidemic take-off (defined as the probability that a primary case generates epidemics which involves at least two other farms) for the entire set of simulated incursions. The probability of epidemic take-off for a single incursion event in GB was 0.028, 0.053, 0.067 and 0.078 for a HRP of 2, 4, 6 and 8 weeks, respectively (Table 1). Furthermore, there was little monthly variation in the probability of epidemic take-off (Fig. 2b).

Under the scenario that detection of an incursion event occurred after eight weeks, we further explored which features of the primary case may influence the risk of spread. Here, the risk of CSF spread was assessed in terms of probability of epidemic take-off for each farm we sampled to be primary case. We quantified a set of six variables which may influence the risk of CSF spread from each of the primary cases (Supplementary Methods S1). These six variables were (1) the producer type of the primary case (i.e. whether it is an assured commercial producer, a non-assured commercial producer, or a small producer, as defined by their population size, movement activity and the quality assurance scheme membership such as in Porphyre *et al*.^17^), (2) the total number of movements departing from the primary case within the study period, (3) the total number of movements arriving into the primary case within the study period, (4) the total number of movements departing from the primary case to a gathering place within the study period, (5) whether a movement from the primary case to another farm were recorded within the study period, and (6) the density of commercial farms at the location of the primary case. From these, independent composite variables were created using the ordination method nonmetric multidimensional scaling (NMS, Supplementary Methods S1). The NMS method identified two dimensions (Supplementary Fig. S1) that explained 90.1% of the variation in CSF risk, with the first and second NMS axis explaining 70.5% and 19.6%, respectively. The first NMS axis was strongly associated with the producer type of the primary case (P < 0.001) and with incursion located in areas where the density of commercial farms is high (Kendall τ = 0.384, P < 0.001), whereas the second NMS axis was associated with producers moving pigs to at least one other farm (P < 0.001, Supplementary Methods S1). Altogether, both NMS axes suggest that incursions in areas with a high density of commercial producers, regardless of whether they move pigs, are more likely to show a high risk of CSF spread (probability of epidemic take-off: Kendall τ = 0.270, P < 0.001). However, incursions into other areas may still generate epidemics (Supplementary Fig. S1).

### Spatial structure and role of producer types

**Figure 3:**
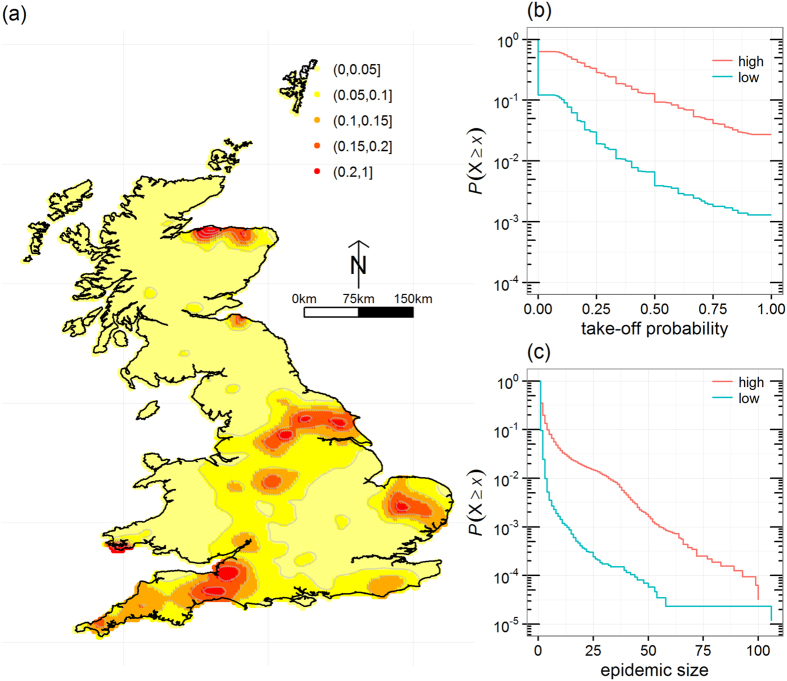
Spatial variation in the risk of CSF spread in Great Britain. (**a**) Smoothed spatial distribution of the probability of epidemic take-off generated from a single incursion. (**b,c**) Decreasing cumulative probability function of (**b**) the probability of epidemic take-off for individual primary cases and (**c**) the epidemic size for incursions occurring in either low or high risk areas. Low and high risk areas are defined as areas in panel (a) where the smoothed probability of epidemic take-off is ≤0.05 and >0.15, respectively. Smoothed probability map in panel (a) was created in R version 3.2.5^48^,^51^ using the kernel intensity ratio method^46^. The probability of epidemic take-off in panels (a,b) is defined as the probability that a primary case generates epidemics which involves at least two other farms. Epidemic size in panel (c) was characterized by the total number of infected farms (excluding primary cases) involved in epidemic generated by individual primary cases. All simulations in these figures considered an eight-week high risk period (HRP). Note that the *y*-axis in panels (b,c) are log_10_-scaled.

To visualise how the risk of CSF may vary spatially, we produced a smoothed map of the farm-level probability of epidemic take-off under the scenario that HRP = 8 weeks. Figure 3a shows distinct areas where the overall CSF risk is greater than the rest of the country. Most of these high risk areas were concentrated in pig-dense areas (Supplementary Fig. S2).

From Fig. 3a, we defined low, medium and high risk areas as locations where the smoothed probability of epidemic take-off is ≤0.05, between 0.05 and 0.15, and >0.15, respectively. To evaluate if the low, medium and high areas differed in their farm population structure and evaluate the influence of small producers in these areas, we first assumed that all farms, regardless of their producer type, are homogeneously distributed. The expected number of farms (E) of each producer type was then compared to the observed numbers of farms (O) in the defined low, medium and high risk areas (Table 2). The density of pig producers in high risk areas (whether commercial or non-commercial) was at least two times greater than expected, with a density of small producers, non-assured commercial producers and assured commercial producers 2.02, 2.70 and 5.4 higher within the high risk areas, respectively. In contrast, low risk areas were characterised by lower densities of farms than expected, with O/E ratio of 0.44 and 0.25 for non-assured and assured commercial producers and 0.61 for small producers.

Figure 3 compares the distributions of the probability of epidemic take-off for all primarily infected farms (Fig. 3b), and the resulting epidemic size (Fig. 3c) for incursions occurring either in high or in low risk areas. Table 1 provides further details on the epidemic features for incursions in each risk area. Although incursions in low risk areas have a much lower chance to generate epidemics, widespread epidemics still occurred from incursions in low risk areas, potentially generating epidemics of more than 100 farms.

To better understand the interactions between risk areas in generating widespread epidemics (defined as those infecting more than 50 farms), we looked at the distribution of infected farms with regards to the risk area they belong to. Over all simulations, 149 incursions generated widespread epidemics, among which 9, 23, 50 and 67 occurred when HRP lasted for 2, 4, 6 and 8 weeks, respectively. These widespread epidemics involved a median of 58 farms (interquartile range IQR: 54–67 farms) and lasted for a median of 181 days (IQR: 142–248 days). Among these 149 widespread epidemics, 9, 24 and 116 were generated from incursions in low, medium and high risk areas. However, most widespread epidemics involved farms from high risk areas. Even on the rare occasions when widespread epidemics did start from incursions in low risk areas, it appears that they did not involve many farms from low risk areas. For example, when only considering widespread epidemics generated from incursions in low and high risk areas, 78% (IQR: 40–78%) and 90% (IQR: 79–96%) of the infected farms were located in high risk areas, respectively. The results are summarized in Supplementary Fig. S3.

**Figure 4:**
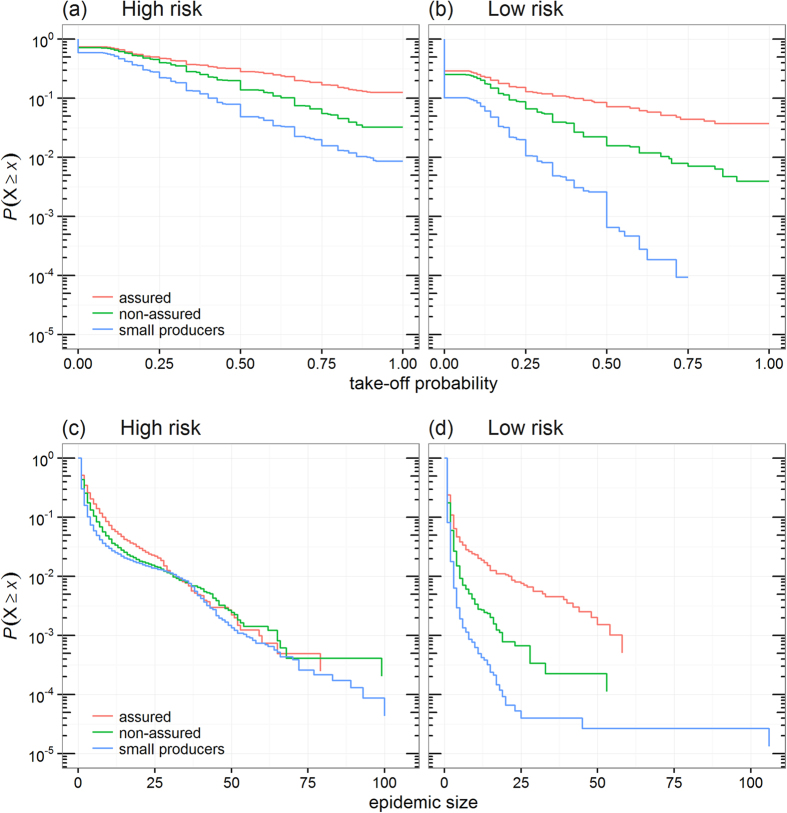
Changes in the risk of CSF spread in Great Britain as a function of producer type and risk areas. Decreasing cumulative probability distribution of (**a,b**) the probability of epidemic take-off for individual primary cases and (**c,d**) the epidemic size generated by a single incursion event occurring in farm of a given producer type and located in either (**a–c**) high and (**b–d**) low risk areas. Low and high risk areas are defined as areas in Fig. 3a where the smoothed probability of epidemic take-off is ≤0.05 and >0.15, respectively. The probability of epidemic take-off in panels (a,b) is defined as the probability that a primary case may infect at least two other farms. Epidemic size in panels (c,d) was characterized by the total number of infected farms (excluding primary cases) involved in epidemic generated by individual primary cases. All simulations in these figures considered an eight-week high risk period (HRP). Note that the *y*-axis in panels are log_10_-scaled.

We looked at how the producer type of the primary case may influence the risk of CSF spread in GB. Figure 2c shows the change of the probability of epidemic take-off in GB for incursions occurring in small producers, non-assured commercial producers and assured commercial producers for the four considered HRP. While the probability of epidemic take-off increased markedly with the duration of the HRP for commercial producers, it remained relatively stable for non-commercial producers (Fig. 2c). Overall, in the situation where HRP = 8 weeks, incursions starting in small producers were four times less likely to generate epidemics than assured commercial producers (Table 1), particularly due to a low probability of infecting other farms. Incursions starting in small producers were also ten times less likely to be detected than assured commercial producers (Table 1). While there was clear division between small producers and commercial producers in the likelihood of generating and detecting epidemics, in both high and low risk areas, the same clear division was not observed for the size of epidemics when incursions occurred in high risk areas. Figure 4 shows the distribution of the probability of epidemic take-off for all primarily infected farms and the distribution of the epidemic size resulting from incursions occurring in the three producer types and for HRP = 8 weeks. Clearly, the overall size of epidemics generated from incursions in high risk areas were not influenced by the producer type of the incursions (Fig. 4C). However, this is not the case when incursions occurred in low risk areas (Fig. 4D).

**Figure 5:**
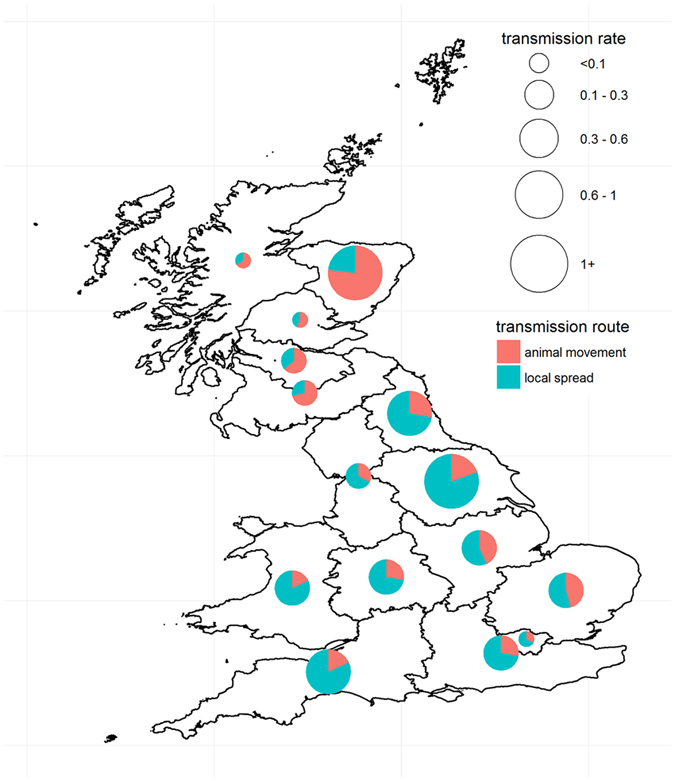
Geographic variations in the transmission of CSF in Great Britain. Pie charts show the proportion of infection events due to the movement of pigs among all infection events between farms recorded in epidemics generated from incursions located in a region of GB. The size of each pie chart further shows the between-farm transmission rate in each region of GB, defined as the average number of infected farms generated from single incursions. All simulations in these figures considered an eight-week high risk period (HRP). Map was created in R version 3.2.5.^48^,^51^.

Assuming that the contribution of transmission routes may differ throughout GB, we looked at the variations of the proportion of infection events due to pig movements compared to those due to local spread for incursions occurring across GB. To this objective, we divided GB into 16 regions, which roughly correspond to the spatial structure of both pigs and pig farm density in GB^17^,^18^, and evaluated, for each region of the incursions, the route of transmission for each infected farm recorded in generated epidemics. Figure 5 shows a clear distinction between Scotland and the rest of GB, with 57% to 77% of infection events in epidemics generated from incursions occurring in the five Scottish regions due to animal movements. In contrast, 54% to 82% of the infection events generated from incursions in England and Wales were due to local spread. Such a pattern was consistent for all HRP scenarios tested (Supplementary Fig. S4). To further explore the relative contribution of movement-based transmission in the spread of CSF in Scotland and England/Wales, we looked at how the odds of movement-based infections may vary between producer types of incursions through a generalized linear regression model (Supplementary Table S1). This simple model not only confirmed that CSF would spread more through animal movements than through local spread from incursions in Scotland (likelihood ratio test statistics P-value P_*LRT*_ < 0.001) but also highlighted that the main transmission route in epidemics may depend on the producer type of the primary case (P_*LRT*_ < 0.001). For incursions occurring in assured, non-assured and small producers from England/Wales, the probability of farms being infected from animal movements would be 0.47, 0.33 and 0.17, respectively. In contrast, the likelihood for CSF to spread through animal movements would be 0.80, 0.72 and 0.46 if incursions occurred in Scotland and in assured, non-assured and small producers, respectively.

### Sensitivity analysis

To identify which aspects of the model most affect the spread of CSF in GB, we calculated the total effect (*D*_*Tk*_) of all *k*^th^ individual model parameters by performing a global sensitivity analysis of the model on the risk of CSF spread. The interest of performing a global sensitivity analysis lies in revealing to what extent uncertainties in parameter values may affect the model response, accounting for both direct effects and those emerging from interactions with other parameters. Here, all parameters involved in the model, namely latent (*T*_lat_) and infectious (*T*_inf_) periods of the virus strain, the basic reproduction number (*R*_0_), the parameters that modulate the height (*k*_1_) and the shape (*k*_2_ and *k*_3_) of the local spread function, the parameters *r*_det_ and *s*_det_ which are involved in estimating the detection time of each infected farms, and the proportion of movements (*c*_*M*_) from small and non-assured commercial producers to escape post-detection restrictions (Table 3), were included in the global sensitivity analysis.

**Figure 6:**
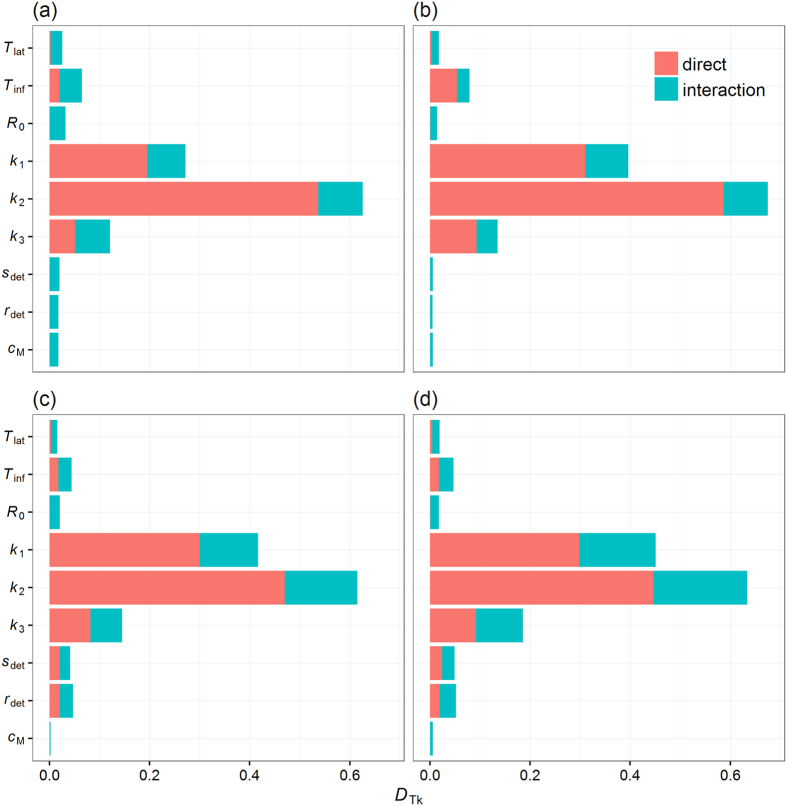
Influence of the model’s parameters on the risk of CSF spread. Results of the global sensitivity analysis on (**a,b**) the probability of epidemic take-off and (**c,d**) maximum epidemic size for single incursion events occurring in either (**a,c**) high and (**b,d**) low risk areas. Influence of the model’s parameters was measured by the total sensitivity index (*D*_*Tk*_), which captures the overall effect of parameter variations, including direct effects and interactions between model parameters. Parameter definitions and range of values considered in the analysis are given in Table 3. Low and high risk areas are defined as areas in Fig. 3a where the smoothed probability of epidemic take-off is ≤0.05 and >0.15, respectively. The probability of epidemic take-off is defined as the probability that a primary case generates epidemics which involves at least two other farms. All simulations in these figures considered an eight-week high risk period (HRP).

Figure 6 shows the influence of all model parameters on the probability of epidemic take-off and the maximum epidemic size for incursions occurring either in low and high risk areas. Varying model parameters shows a limited impact on both epidemic outcomes, with relatively constant *D*_*Tk*_ values and ranks for each parameters regardless of the location of the primary case (Supplementary Table S2). Whether incursion occurred in high or low risk areas, uncertainties in the local spread parameters (i.e. *k*_1_, *k*_2_, *k*_3_) have the greatest impact on the model outcomes, with *D*_*Tk*_ values >0.5 for all these parameters. Beside local spread parameters, the probability of epidemic take-off is further marginally affected by variations in the length of infectiousness duration *T*_inf_ (*D*_*Tk*_ = 0.07), whereas variations in *T*_inf_ and *r*_det_ marginally affected the maximum epidemic size (*D*_*Tk*_ ~ 0.05). There is a clear distinction between incursions occurring in low risk areas and those occurring in high risk areas with respect to median epidemic size and the proportion of infection due to animal movements (Supplementary Fig. S5). Although *k*_1_, *k*_2_, *k*_3_ remain the most influential parameters, *T*_inf_, *T*_lat_, *r*_det_ and *s*_det_ show a greater influence (with *D*_*Tk*_ values >0.1) on the median size of epidemics when incursions occurred in a low risk area than when they occurred in a high risk area. This finding highlights the importance of both the strain of the virus and the time taken for detecting disease during outbreaks when incursions occurring in low risk area. Interestingly, while *c*_*M*_ (the level of compliance of post-detection movement restrictions by small and non-assured producers) was the least influential parameters when considering the probability of epidemic take-off, the maximum epidemic size or the median epidemic size, its variations showed an important influence (*D*_*Tk*_ = 0.29) on the contribution of transmission via animal movements in low risk areas, ranking fourth most influential parameter behind *k*_2_, *k*_3_ and *s*_det_ (Supplementary Table S2 and Fig. S5). However, 90% of this influence is in interaction with other parameters (1 − *D*_*k*_/*D*_*Tk*_ = 0.91, Supplementary Table S2), mostly with *k*_1_, *k*_2_ and *k*_3_.

## Discussion

For pathogens that are likely to spread rapidly within and between farms, the vulnerability of an industry can be measured by how susceptible the industry is to suffer severe epidemics if incursion occurs. In this study, we have assessed the risk of CSF spread within the British pig industry, in the event that the virus is introduced anywhere in GB. We have shown that, although unlikely, widespread epidemics of CSF could happen at any time of year in GB, regardless the duration of the high risk period. This was accomplished by using data from an historical outbreak in East Anglia to parameterise a stochastic model, thus combining representative epidemiological information with the large-scale transmission between premises in GB and a detailed description of the within-farm dynamics of infection and disease. Using this model, we further explored the resilience of our estimate of the risk of CSF spread to the various uncertainties which fundamentally underlie the stochastic nature of both disease incursion and control activities currently defined by the UK contingency plan against CSF^19^. These are particularly relevant to the improvement of preparedness for disease incursion and provide valuable stepping stones in guiding policy on CSF surveillance and control in GB.

The potential spatial extent and size of an epidemic largely depends on the number of farms that have already been infected at the moment the disease is first diagnosed^20^. Rapidly detecting disease (or in other words, minimising the duration of the HRP) is therefore of great importance in limiting the size of an outbreak. We have shown in this study that even if efforts are made to minimise the HRP to four weeks, which would be challenging, only a quarter of CSF introduction events in GB would be detected (Table 2). Although efforts have been made to increase the awareness and vigilance of farmers and veterinary services, surveillance activities are still based on diagnosing CSF from clinical signs of diseased animals^21^, which are difficult to detect with certainty, particularly at the early stage of infection^22^ and outside outbreak periods^23^. There is also significant variation in clinical presentation between different CSF strains, and between different animals groups, since clinical signs depend on age, breed and immune status of the infected animal^1^. Given the impact that the duration of the HRP has on the likelihood of severe disease outbreaks, additional efforts to ensure that disease incursions are quickly detected would be worthwhile. Risk-based surveillance has been advocated to promote early detection, targeting either production type (e.g. breeder, finisher, weaner-breeder)^20^,^24^,^25^, or farms with high mortality records^15^. In this study, we evaluated where and when CSF would be more likely to spread, assuming a long HRP, as a basis for improving surveillance activities. Indeed, prioritising areas of higher risk of disease spread is critical for surveillance planning and resource allocation, enabling veterinary services to detect and respond much faster to disease introduction.

Under the extreme (but not unlikely) scenario that eight weeks passes before an incursion is detected, there was a clear spatial structure in the risk of disease spread in GB (Fig. 3a). Most high risk areas are concordant with locations with a high density of pigs (Supplementary Fig. S2), indicative of the location of industry hotspots. While this finding is in agreement with the outcome of the NMS analysis, suggesting that high density of commercial farms is a key driver in the risk of CSF spread, high risk areas are not only limited to industry hotspots. Comparing the map of high risk of CSF spread (Fig. 3a) with the distribution of pigs and pig farms in GB (Supplementary Fig. S2) shows that some risk areas are located in areas where the density of pigs is low but the density of pig farms is high. Together with the fact that twice as many small producers are located in high risk areas than expected (Table 2), these findings suggest that areas with a high density of small producers may also generate large epidemics.

There is little research on the role of small producers, which in GB predominantly includes backyard holdings and traditional small-scale farms, in the spread of notifiable disease in Europe. Small producers are usually regarded as having little impact on the risk of CSF spread, only participating in the persistence of diseases in the industry^26^. Such a hypothesis has been supported by modelling work on the spread of CSF in Bulgaria, which suggested that incursion in backyard producers would be unlikely to infect the commercial sector of the industry^27^. Our results suggest, however, that the risk of CSF spread in GB when incursion occurs in small producers is potentially not negligible, and that incursions could generate widespread epidemics (Fig. 4 and Table 1). This is a serious concern because small producers are less knowledgeable about biosecurity and disease risks. For example, a quarter of surveyed backyard farmers in England reported feeding household scraps^28^, a practice that can lead to the initial incursion of swine diseases^29^,^30^,^31^,^32^ and which is banned in the EU (Regulation (EC) 1069/2009). In addition, a substantial proportion of pig farmers in Europe were found likely to not report, or at least not quickly enough, clinical signs of swine fevers^33^,^34^, and the current routine surveillance systems would not (quickly) detect incursions in the non-commercial sector of the industry^25^. These factors highlight the importance of ensuring surveillance activities include small producers as well as communication efforts to raise awareness of diseases and best practices in this sector of the industry.

Of particular interest, is the high risk area found in the South West of England. It has been previously reported that this region has a large number of outdoor backyard pig producers together with increasing populations of wild boars^29^. Contacts with the wild boar population (either direct or indirect) are regarded as important determinants in the persistence and spread of swine fevers in Europe^1^. Although it is not clear if the population density in this wild boar population is high enough for disease to persist, this suggests nevertheless that our model, which did not include wild boar, may have underestimated the risk of CSF spread.

In addition to their timing and location, incursions may be characterised by other features that present various levels of uncertainty, and which may impact on the risk of CSF spread. In this paper, we examined the impact of uncertainties in virus characteristics (as informed by *T*_lat_, *T*_inf_ and *R*_0_), local between-farm spread (*k*_1_, *k*_2_, *k*_3_), surveillance activities (*r*_det_, *s*_det_) and human behaviour (*c*_M_) on the risk of CSF to spread in the industry. Over all parameters involved in the model, varying the values of those involved in the local spread showed the greatest impact on the risk of CSF spread. This finding is consistent with other simulation outputs looking at the spread of foot-and-mouth disease in North America^35^ and in the UK^7^,^10^, showing that variations in the width and height of the transmission kernel function would significantly impact on the number of infected premises. It is therefore clear from our finding that using function of local spread fitted from outbreaks occurring elsewhere in Europe would have significantly biased our inferences. Comparing the transmission kernel function from GB with that from the Netherlands^15^ showed that the local between-farm spread in GB is significantly lower at short distances (up to 4 km) than in the Netherlands (Fig. 1a). Using the shape of the local spread fitted from the Netherlands situation would have therefore considerably overestimated the risk of CSF spread in GB.

The local between-farm transmission of highly infectious diseases is generally due to multiple factors related to human behaviour and movements of fomites. In the case of CSF, transmission by infected rodents and pets, and by contaminated vehicles have been shown experimentally to be unlikely^36^,^37^, whereas indirect transmission via people can occur^38^. The high sensitivity of the risk of CSF spread to variations in the local between-farm spread parameters (i.e. *k*_1_, *k*_2_, *k*_3_) confirms therefore that ensuring limited local spread is critical to prevent widespread epidemics in GB. Encouraging high standards of between-farm biosecurity (whether prior or post disease incursion) is therefore of utmost importance. Pre-emptive depopulation of farms within the vicinity of IPs, although another way of achieving control of local spread, is unlikely to be implemented in the UK during an outbreak of swine fever (The Diseases of Swine Regulations 2014, S.I. 2014/1894). However, these results are based on the assumptions that local spread is fixed, regardless of the local farm density, of the type of infected producers or the number of infectious individuals present in the farm, and active as soon as farm are infected. Such assumptions may overestimate our inferences on the local spread of CSF in GB, particularly in areas where backyard and non-commercial farming prevails. Indeed, in this study, we used the data from the 2000 CSF outbreaks in which only commercial pig farms were found infected with CSF^6^. Given that trading and biosecurity behaviours differ between commercial and non-commercial farms^17^,^28^,^33^,^34^,^39^, it is likely that the kernel shape would differ even after movement restrictions and enhanced biosecurity measures were implemented (as a response to the detection of the first outbreak farm). However, at the time of writing there is no published literature on the influence of producer types on the shape of transmission kernel function. Although Boender *et al*.^14^ showed that the number of animals on the farm would significantly affect the shape of the transmission kernel function, their analysis included only commercial farms with high level of biosecurity, which is not the case in backyard farms^28^,^40^. Considering the sensitivity of the model to the parameters of the transmission kernel function, further work is required to better understand how the shape of this kernel may vary between sectors of the industry (including small producers) if we are to accurately predict the spread of the disease.

Once disease is detected, movement restrictions form part of the disease control strategy. Previous studies on compliance with movement restrictions suggested that decreasing compliance would drastically affect the spread of disease^11^,^12^. Our model assumed that industry incentives to respect regulations and implement biosecurity standards would ensure optimum compliance from assured producers in GB, whereas other producer types may be less compliant. Such a hypothesis was chosen to evaluate where efforts should be carried out during an outbreak, particularly with regards to enforcing movement restrictions on a large, poorly characterised population such as the backyard population. Our study clearly showed that varying compliance to post-detection movement restrictions of producers who do not belong to assurance scheme did not significantly influence the risk of CSF spread in GB. Whether it is because movements from/to small producers are infrequent and involve small number of pigs per batch^17^ or because many of the moves involving small pig keepers also involve assured producers, and hence would be stopped anyway, is unclear. However, these findings indicate that rather than enforcing 100% compliance to movement restrictions on all producers across GB, it would be more cost-efficient to advise movement restrictions to all producers but ensure 100% compliance on all assured producers.

In conclusion, this study provides insight on how notifiable diseases such as CSF could spread within the British swine industry. We found that large outbreaks of CSF would be rare and generated from a limited number of areas in GB. Although our results confirm that the British swine industry has a low vulnerability to large CSF outbreaks, we identified concerns with respect to the role played by the non-commercial sector of the industry. This study has highlighted several areas where efforts could be made to improve preparedness for CSF incursions in the British swine industry. Particularly, further work is required to better understand the role of small pig producers in GB in the spread of notifiable diseases and how local spread would be affected by their behaviours.

## Material and Methods

The spread of CSF within the British swine industry was modelled using a hybrid stochastic-deterministic, spatially explicit, premises-based, discrete-time model where within-farm prevalence is modelled explicitly. The model comprises modules which account for (1) the transmission of the disease between premises, (2) the influence of the within-farm prevalence on disease transmission between premises, and (3) mitigation and surveillance activities carried out to control epidemics.

### Model structure

The model implicitly follows a Susceptible-Infected-Recovered epidemiological process, in which infection is transmitted from an infected (and infectous) premises (state *I*) to susceptible premises (state *S*) with a probability *P*_*i*_, and in which infected premises remain infectious for a period of time until they recover and become immune to further infection (state *R*). Premises enter the immune state in two ways: either (1) they are detected and reported as infected to animal health agencies and therefore control activities are carried out, or (2) they are not detected, and disease progresses within premises until all pigs are either dead or immune.

In addition to swine producers, gathering places (i.e., markets, show grounds, ferry collection centres and slaughterhouses) were included in the model and could contribute to the spread of CSF. As in most EU countries, regulations are in place in GB to limit the spread of pathogens via the movements of animals through gathering places. Gathering places are not permitted to keep pigs overnight and are required to implement cleaning and disinfection procedures after each day of activity (The Animal Gatherings Order 2010, S.I. 2010/460). As such, the model assumes that all infected gathering places would go back to the susceptible state after one day (thereby following a Susceptible-Infected-Susceptible process). For the purpose of this study, we defined “premises” as all pig farms (also named swine producers) and gathering places present in GB during the study period.

The model is seeded at incursion time *t*_0_, progresses in discrete time steps Δ*t* of one day, and runs until no infectious premises remain present in the industry.

### Transmission between premises

The model assumes that each *i*^*th*^ susceptible premises would be infected by an infectious premises *j* with a daily probability *P*_*i*_ depending on (1) the number of animals that have been received from premises *j*, (2) the animal-level disease prevalence *w*_*j,t*_ that occurs in premises *j* at the time *t* movement is carried out, and (3) its spatial location, relative to that of infected premises *j*. However, because the order in which different transmission processes occur may have some impact on the rate of disease spread, we assumed that movements to gathering places would be carried out first at the start of every simulation day, followed by movements to other premises, followed finally by transmission through local spread at the end of the simulation day. As such, we separated the daily probability of premises to be infected due to local spread *L*_*ji,t*_ to that due to the movements of pigs *M*_*ji,t*_ such as


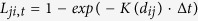


and


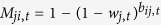


where *b*_*ij,t*_ is the number of pigs that have been received by the premises *i* from any infected premises *j* at time *t*. Values of *b*_*ij,t*_ were extracted from historical records of pig movements that occurred in Great Britain from January 1^st^ 2012 to May 31^th^ 2013. The component *K(d*_*ij*_) of equation (1) denotes the “between-farm transmission kernel function” and determines the rate at which an infected premises may infect susceptible ones as a function of inter-farm Euclidean distance *d*_*ij*_. *K(d*_*ij*_) describes the spread that results from local transmission dynamics that are independent from the movements of animals. This encompasses several routes of infection, including travel of farm workers, sharing farm materials or other locally driven processes linking premises, which are likely to occur more often between spatially-close farms than between farms separated by longer distances. Finally, the model consider that the producer type of the source premises *j* in equation (2) did not influence the disease transmission dynamics: that is, all premises were assumed to be identical in this respect.

### Transmission within premises

The dynamics of the infection within premises determine what proportion of the total number of pigs in premises is infectious at each time step and how rapidly infected animals will recover. This, in combination with the number and size of batches of pigs moved from premises, will determine what proportion of movements from infected premises will contain infected individuals (Eq. 2).

When disease is introduced, the spread of the disease within each premises *i* was modelled using a deterministic, frequency-dependent model. Following exposure, a susceptible (*S*_*h*_) animal becomes exposed (*E*_*h*_) with a mean latent period *T*_*lat*_, after which it will be infectious (*I*_*h*_) for a mean infectious period *T*_inf_ until it recovers or dies (*R*_*h*_). In the model, the variables *S*_*h*_, *E*_*h*_, *I*_*h*_, and *R*_*h*_ represent the number of pigs in premises in each epidemiological stage. The prevalence *w*_*i*_ is therefore computed as the proportion of pigs in premises that are in the infected states (whether infectious or not), such as *w*_*i,t*_ = (*E*_*hi,t*_* + I*_*hi,t*_)/*N*_*i*_ where *N*_*i*_ is the total number of pigs in premises.

Considering a closed population of size *N*_*i*_, we take transmission of CSF within each *i*^*th*^ premises to be frequency-dependent, with an effective transmission rate *λ*_*i,t*_ = *βS*_*hi,t*_/*N*_*i*_where *β* is the within-premises transmission rate. Here, frequency-dependence was considered as we expect the number of contacts between pigs to be independent of the size of the pig population within each premises, in compliance with herds stocking densities regulations^41^. The transmission rate *β* was derived from the basic reproduction number *R*_0_ (the mean number of secondary cases generated by each infected individual in a totally susceptible population) as extracted from Backer *et al*.^15^.

As the duration of infectiousness may not decay exponentially^15^, *T*_inf_ was modelled decomposed in a sequence of 10 independent stages, corresponding to an Erlang distribution with mean 15 and shape parameter 10.

### Mitigation procedures

Mitigation procedures implemented in the model were those defined by the UK Contingency plan for CSF^19^. Pre-emptive depopulation of pig farms solely based on the distance to infected premises as well as the implementation of a vaccination strategy are therefore not considered in this study. Instead, when infected premises are detected, whole farm depopulation measures are implemented within 24 hours and a 10km-radius surveillance zone (SZ) is enforced around the location of each detected infected premises.

Movements on to- and off of all premises located within each SZ, whether those premises are known to be infected or not, are prohibited. As previous outbreaks showed that the efficiency of movement restrictions may not be perfect^42^, the model allows a pre-defined proportion of movements *c*_*M*_ to continue despite restrictions. In reality, full compliance is expected from commercial producers belonging to an assurance scheme. Therefore, our model assumes that compliance in movement restrictions would only matter for producers that are not members of an assurance scheme. However, unless otherwise stated, we considered a baseline model configuration which assumes no variation in compliance in movement restrictions (*c*_*M*_ = 1).

Although the detection of CSF on infected premises is a multifactorial process, depending, for example, on the virulence of the CSF strain, the report (or not) of previously infected premises in the country and the intensity of surveillance activities, we assumed that the detection time *T*_det_ of each infected premises was dependent only on the time since infection^15^,^43^ and the presence of previous reports of CSF in the country. Once CSF has been detected (i.e. after the HRP has passed), the value of *T*_det_ was modelled as a left-truncated Gamma distribution, with a minimum detection time of two latent periods (i.e. 2*T*_lat_ = 8 days)^15^, with a rate *r*_det_ and a shape parameter *s*_det_ randomly allocated for each infected premises. In reality, surveillance activities are imperfect and may miss detecting infected premises. As such, when the within-farm spread has ended (i.e., when less than one infectious or exposed individual remains) prior to the assumed detection time, infection will go unnoticed. For simplification, infected gathering places were set to have a fixed probability of detection, currently assumed being equal to zero.

### Estimation of model parameters

In total, the model includes 10 parameters (Table 3). Parameter values used to inform the within-premises transmission component of the model were taken from published literature (Table 3), whereas the shape of the transmission kernel function *K(d*_*ij*_) as well as the rate *r*_det_ and shape *s*_det_ of the detection function were fitted together using data from the CSF epidemic in East Anglia, UK that occurred in 2000^6^. The data includes information regarding the spatial location and the time of report for all premises (*n* = 16 farms) for which CSF have been reported between August 8^th^ to November 3^rd^ 2000 and for which mitigation procedures were carried out.

Because only 16 cases were reported^6^ and because the infection times of reported infected premises were uncertain, a Bayesian framework approach^44^ was used to fit the joint transmission and detection processes. Briefly, we considered a similar epidemiological process as the simulation model, with the spread of the epidemic between farms modelled using a stochastic and spatial Susceptible-Infected-Recovered model in which the transmission kernel function *K(d*_ij_) is parameterised as in Boender *et al*.^45^:


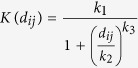


where *k*_1_, *k*_2_ and *k*_3_ are the fitted parameters.

Markov Chain Monte Carlo (MCMC) method was used to draw samples from uninformative prior distributions, with sampling framework based on the Metropolis-Hastings and Gibbs sampling algorithms. Details on the Bayesian inference method to estimate model parameter values are provided in Supplementary Methods S2. The kernel parameters found for the CSF epidemics in East Anglia were *k*_1_ = 0.0007249 day^−1^ (95% Credible Interval (Cr.I.) 0.00003811–0.005492), *k*_2_ = 0.27859 km (95% Cr.I. 0.04077–5.531453), *k*_3_ = 1.7098 (95% Cr.I. 0.9411–3.7957), whereas the detection function parameters were *r*_det_ = 0.1286 day^−1^ (95% Cr.I. 0.04991–0.2892) and shape *s*_det_ = 4.3827 (95% Cr.I. 1.9956–8.7009) (Table 3 and Fig. 1). Based on the fitted parameters of the detection function, a median of 31.9 days (95% range: 11.1–71.7 days) after infection was therefore required to detect infected farms in our simulations.

### Data collection and population at risk

All premises in GB recorded as having pigs on site or recorded as participating in the movement of pigs from January 1^st^ 2012 to May 31^st^ 2013 were included in the study and were identified by their unique county/parish/holding (CPH) identifier. Records for the presence of pigs on farms were taken from the Scottish Agricultural and Horticultural Census (2011 to 2013), English Survey of Agriculture, Horticulture, and Labour (2010) and the Survey of Agriculture and Horticulture (2011 to 2012), the Welsh Agricultural & Horticultural Survey (2010), and the Quality Meat Scotland (QMS) and Red Tractor registers for 2013. All movement data were extracted from the Scottish livestock electronic identification and traceability database (ScotEID)^17^ and the electronic Movement Licensing database (eAML2). All premises with an unknown CPH identifier from these databases were cross-checked against the 2013 pig keeper register, which records all holdings keeping pigs in GB and is owned by the Animal & Plant Health Agency (APHA). In order to not exclude (most likely non-commercial) producers from our study, we further included in the analysis premises that were reported with an unknown CPH identifier (either as a destination or a departure of a movement) if there was a corresponding valid postcode. Details of spatial location for premises were extracted from the eAML2 location data, the ScotEID postcode address field, the coordinates from the agricultural surveys for 2010, the QMS and Red Tractor registers, or the pig keeper register.

Together, ScotEID and eAML2 databases provide a comprehensive picture of all displacement of pigs in GB at the batch rather than individual pig level. Each movement record reports the CPH for both departure and destination premises, the number of animals moved, and the date on which the movement occurred. Details of premises type for departures and destinations are also recorded in the movement databases, allowing slaughterhouses, markets, show-grounds and collection centres to be differentiated from agricultural holdings. In this study, all farms were classified into three group types of producer as previously defined^17^. Briefly, pig producers were classified according to their population size, movement activity and the quality assurance scheme membership:

1. “Small pig producers”: agricultural holdings with an unknown number of pigs; or less than five sows, and/or less than 10 finishers; and showing no records of movements of more than 50 pigs within the study period.
2. “Non-assured commercial producers”: agricultural holdings with more than five sows and/or more than 10 finishers; or showing records of movements of more than 50 pigs during the study period, but that do not belong to a quality assurance scheme from QMS or Red Tractor, the main British assurance schemes.
3. “Assured commercial producers”: agricultural holdings with more than five sows and/or more than 10 finishers; or showing records of movements with more than 50 pigs during the study period belonging to a quality assurance scheme from QMS and/or Red Tractor.

In total, this study comprises 429 gathering places and 34,294 farms of which at least 1 pig were recorded moving or present in GB. Among these 34,294 farms, 5.4% were assured commercial producers, 13.1% were non-assured commercial producers and 81.5% were classified as being small pig producers (Table 2).

Over the 34,294 identified pig farms, 23,555 did not show any records of population size in the considered databases. Population size for farms where this information was not reported were inferred based on information recorded in the most recent agricultural census as a function of their producer type. Records from assured commercial producers, non-assured commercial producers and small pig producers showed a median population size of 1450 (Q1–Q3: 783–2738), 49 (Q1–Q3: 19–285) and 3 (Q1–Q3: 2–5) animals, respectively. For the purpose of this study, assured commercial producers, non-assured commercial producers and small pig producers with unknown population size were therefore considered to keep 1450, 49 and 3 pigs on farm, respectively.

### Model implementation

The model assumes that an incursion of CSF in GB occurs in a single pig farm and that the primary case is infected by introduction of a single (infected) animal. In the absence of information about where CSF incursion is most likely, we assumed that all pig farms would have the same probability of being infected by CSF and becoming a primary case, regardless of their spatial location or their production type. All primary cases used in this study were therefore randomly chosen from the list of available pig farms. Within the period January 1^st^ 2012 to December 31^st^ 2012, 20,000 randomly-allocated incursions were generated in the first Monday of each month, resulting in 240,000 model runs.

When modelling local spread, computing the probability of premises being infected across GB at each time step via local spread proved prohibitively time consuming when using the full list of premises with pigs in GB. We therefore reduced the computational burden by considering that infected premises would be able to transmit CSF only to susceptible premises within a 20 km radius area via local spread. As such, all premises beyond 20 km of the nearest infected premises were assumed to escape infection via local spread. Such a distance (i.e. 20 km) was determined to represent twice the distance enforced in the UK for implementing movement restrictions around IPs (i.e. 10 km). Recent studies on the movement of pigs in both England^39^ and Scotland^17^ further showed that movement of pigs from commercial farms would mostly travel beyond 20 km. As such, using this approximation further avoids including infections that may results from both animal movements and local spread together.

Each generated outbreak would remain unnoticed for the pre-determined duration of the high risk period (HRP, the length of time that CSF may spread before detection^16^). If more than one premises remains infected after the HRP, a single infected premises is randomly selected as the primary reported case. All mitigation procedures are initiated with 24 hours of the first report and remain active until no infectious cases are present in the population.

### Statistical analysis

Here, we first define the likelihood that an epidemic may be generated from a single incursion event in GB as the probability of epidemic take-off, i.e. the probability that a primary case generates epidemics which involves at least two other farms. This probability was computed by calculating the proportion of incursion events in each individual farm that generated epidemics of more than two farms (including the primary case).

To explore how the risk of CSF spread varies throughout GB and visually compare it with the spatial distribution of pigs and pig farms, smoothed maps of the farm-level probability of epidemic take-off, as well as that of animal density and farm density, were plotted using the weighted kernel intensity ratio method^46^. All intensity surfaces were constructed using a kernel smoothing algorithm that included a correction term for edge effects in the package SPATSTAT^47^ in the statistical software R version 3.2.5^48^. The bandwidth parameter for the kernel functions used to control the degree of smoothing of the estimated intensity surface was arbitrary fixed to 10 km to comply with the regulated size of the SZ in which movement restrictions are enforced in the UK.

A subset of generated outbreaks were analysed to identify factors associated with the probability of epidemic take-off for individual primary cases. Details on data management and statistical analyses are provided in Supplementary Methods S1. Briefly, analysis was carried out using the Nonmetric Multidimensional Scaling (NMS) method. NMS is a nonparametric ordination technique that avoids the assumption of linear relationships among variables by ranking distances between variables. Therefore, it is well suited to data that are non-normal or on arbitrary or discontinuous scales^49^. We used PC-ORD software version 6.08 (MJM software Design, Gleneden Beach, OR) and computed distances between variables using a Euclidian distance measure.

### Sensitivity analysis

To explore the sensitivity of the model to variations in parameters, sensitivity analysis was conducted by computing the total sensitivity index *D*_*Tk*_ using the extension of Fourier amplitude sensitivity test (FAST)^50^. The extended FAST method is a variance-based, global sensitivity analysis technique that quantifies the sensitivity of the model output with respect to variations in each input parameter by means of spectral analysis independently of any assumption about the model structure (such as linearity, monotonicity and additivity of the relationship between input factors and model output). As such, it captures the overall effect of parameter variations, including direct and joint (i.e. interactions between model parameters) effects. In addition to the total effect (*D*_*Tk*_) of all *k* individual parameters, the proportion *D*_*k*_/*D*_*Tk*_ was computed to inform the fraction of *D*_*Tk*_ attributable to the direct effect, *D*_*k*_. Here, a value of zero indicates that the model outcome of interest is not affected by variations of a given parameters. By contrast, values of *D*_*Tk*_ close to one indicate a strong dependence of the model outcomes on parameter variations.

The sensitivity of model outcomes to nine parameters involved in the model (*R*_0_, *T*_inf_, *T*_lat_, *r*_det_, *s*_det_, *k*_1_, *k*_2_, *k*_3,_ *c*_*M*_) was evaluated. For fitted parameters, values were allowed to vary uniformly within the space encompassing their 95% credible intervals. For other parameters, values were allowed to vary uniformly within the range of empirical observations (Table 3). In total, 1800 independent randomly-generated sets of model parameters were used in the global sensitivity analysis.

The model outcomes that we considered were the probability of epidemic take-off (i.e., the probability that a primary case may infect at least two other farms), the maximum epidemic size, and the median epidemic size. All outcomes were computed over 200 independent simulations, for which we considered a single infection event of CSF that was left freely spreading for 8 weeks prior to be detected and post-detection control and surveillance procedures activated. To evaluate if the sensitivity of the model outputs to variations in parameters differs whether incursion occur in a low risk area or in a high risk area, all producers that were found likely to generate epidemics in the NMS analysis and were located in either a low or high risk area were eligible to be a primary case. For simplicity, however, we restricted infection events in each area to 10 farms that were randomly sampled from the list of farms identified in the NMS analysis. In addition, we restricted infection events to occur the first Monday of 2012. Overall, the sensitivity analysis included 720,000 simulations.

## Additional Information

**How to cite this article**: Porphyre, T. *et al*. Vulnerability of the British swine industry to classical swine fever. *Sci. Rep.* **7**, 42992; doi: 10.1038/srep42992 (2017).

**Publisher's note:** Springer Nature remains neutral with regard to jurisdictional claims in published maps and institutional affiliations.

## Supplementary Material

Supplementary Information

## Figures and Tables

**Table 1:** Epidemic impact of CSF incursions occurring in GB.

	*P*_prim_	*P*_det_	*P*_takeoff_	Epidemic size (IPs)^b^		Epidemic duration (days)^b^		
				Median (95%)	Max.	Median (95%)	Max.	%mov
HRP								
2 weeks	0.913	1.000	0.028	1 (1–3)	102	1 (1–30)	356	0.212
4 weeks	0.849	0.150	0.053	2 (1–12)	100	15 (2–88)	>365	0.240
6 weeks	0.825	0.085	0.067	3 (1–25)	113	20 (1–105)	350	0.258
8 weeks	0.813	0.080	0.078	3 (1–30)	107	21 (1–109)	345	0.285
Risk areas^a^								
High	0.651	0.195	0.198	4 (2–40)	101	27 (2–132)	303	0.228
Medium	0.790	0.087	0.085	3 (1–17)	92	19 (1–93)	345	0.305
low	0.905	0.024	0.024	2 (1–15)	107	15 (1–87)	280	0.412
Farm types^a^								
Small producers	0.845	0.032	0.057	5 (2–38)	107	22 (1–112)	345	0.171
Non assured	0.705	0.254	0.141	3 (1–17)	100	18 (1–91)	280	0.363
Assured	0.593	0.392	0.244	3 (1–27)	80	25 (2–122)	274	0.567

*P*_prim_: Probability of epidemics limited to the primary case only; *P*_det_: Probability of detecting incursions; *P*_takeoff_: probability of epidemic take-off; %mov: proportion of infection events due to the movements of pigs; 95%: 95% range.

^a^Computed considering a high risk period of HRP = 8weeks.

^b^Computed for epidemics that lasted more than the pre-determined high risk period.

**Table 2:** Number of pig producers involved in each risk area of CSF spread, stratified by producer type.

Risk areas^a^	Surface area	Total	Small producers (O/E)^b^	Non assured producers (O/E)^b^	Assured producers (O/E)^b^
High	13,638 km^2^	4561	3276 (2.02)	706 (2.70)	579 (5.37)
Medium	73,521 km^2^	17,409	13,905 (1.59)	2521 (1.79)	983 (1.69)
Low	147,671 km^2^	12,324	10,763 (0.61)	1268 (0.44)	293 (0.25)
Total	234,830 km^2^	34,294	27,944	4495	1855

The ratio O/E is the ratio of the observed (O) over the expected (E) number of pig farms per km^2^ in each risk area of CSF spread. The expected number of farms per producer type was computed considering homogenous spatial distribution of farms and proportional to the surface (in km^2^) of each risk area. For example, the expectation for small producers in low risk areas is E = 27,944 * 147,671/234,830 = 17,572 farms.km^−2^.

^a^Low, medium and high risk areas are defined as areas in Fig. 3a where the smoothed probability of epidemic take-off is ≤0.05, between 0.05 and 0.15, and >0.15, respectively.

^b^Producer types are as defined in the text.

**Table 3:** Parameters involved in the transmission model for CSF in Great Britain.

Parameter	Description	Derivation	Values	Sensitivity analysis range
*R*_0_	Basic reproduction number	After Backer *et al*.^15^	2.8	1–15
*T*_lat_	Latent period: period between infection and viremia within the animal	After Backer *et al*.^15^	4 days	2–10
*T*_inf_	Infectious period: period of infectiousness of an animal	After Backer *et al*.^15^	15 days	7–25
*r*_det_	Constant detection rate	Fitted	0.1286 day^−1^	0.04–0.30
*s*_det_	Shape parameter of the detection function	Fitted	4.3827	2.0–9.0
*k*_1_	Kernel parameter	Fitted	7.25 × 10^−4^ day^−1^	4 × 10^−5^–6 × 10^−3^
*k*_2_	Kernel parameter	Fitted	0.2786 km	0.04–5.50
*k*_3_	Kernel parameter	Fitted	1.7098	0.9–4.0
*c*_*M*_	Probability of complying to movement restrictions for farms with no health quality assurance membership	Assumed	1.0	0.5–1.0
