# Supplementary material for: Vulnerability of the British swine industry to classical swine fever
Source: Sci Rep. 2017 Feb 22;7:42992. doi: 10.1038/srep42992 (PMC5320472; doi:10.1038/srep42992)
Supplement: Supplementary Information [file srep42992-s1.pdf]

Vulnerability of the British swine industry to classical swine fever

Thibaud Porphyre[1], Carla Correia-Gomes[2], Margo E. Chase-Topping[1], Kokouvi Gamado[3], Harriet K. Auty[2], Ian Hutchinson[2], Aaron Reeves[2], George J. Gunn[2], Mark E. J. Woolhouse[1]

[1] Epidemiology Research Group, Centre for Immunity, Infection and Evolution, University of Edinburgh, Edinburgh, Scotland

[2] Epidemiology Research Unit, Future Farming Systems, Scotland's Rural College, Inverness, Scotland

[3] Biomathematics & Statistics Scotland, Edinburgh, Scotland

Supplementary Methods

Supplementary Methods S1. Details on the data, method, and results for the nonmetric multidimensional scaling (NMS) analysis looking at factors correlated with the risk of CSF spread in Great Britain.

Data Management

Table A1 (Supplementary Methods S1) contains a list of the six variables included in the analysis. Examination of the structure of the data in Table A1 (Supplementary Methods S1) required that some variables be categorised for the purpose of statistical analysis and others transformed. Table A1 includes a description of how the variables were treated for the purpose of statistical analysis. To limit biases due to farms that underwent few incursion events, we restricted our analysis to farms that had at least 10 incursion events (*n*=5613). From those farms a random sample of 5000 farms were selected. This was done to reduce the computational burden. No dissimilarities were observed

between the reduced and full set of farms for both the distribution of the probability of epidemic take-off and the distribution of maximum epidemic size (Fig. A1 in Supplementary Methods S1).

Table A1. The six variables included in the nonmetric multidimensional scaling (NMS) analysis, including a description of how they were treated in the NMS analysis.

Variable	Data type	Description
Producer type of the primary case	Categorical	Small=1; Non-assured=2; Assured=3
Total number of movements departing from the primary case	Categorical	5 levels: 0=1; 0-1=2; 1-2=3; 2-7=4; >7=5
Total number of movements sent to the primary case	Categorical	4 levels: 0=1; 0-1=2; 1-2=3; >2=4
Total number of movements sent by the primary case to a gathering place	Categorical	5 levels: 0=1; 0-1=2; 1-2=3; 2-6=4; >6=5
Total number of movements sent by the primary case to another swine producer	Categorical	2 levels: 0=0; >0=1
Density of commercial farms	Quantitative	SQRT transformed density commercial

Statistical Analysis

The data in this study were analysed using Nonmetric Multidimensional Scaling (NMS). The NMS method was used to identify epidemiological variables correlated with the risk of CSF spread as assessed in terms of probability of epidemic take-off and maximum epidemic size. NMS is a nonparametric ordination technique well suited to data that are non-normal or on arbitrary or discontinuous scales[1]. The advantage of NMS is it avoids the assumption of linear relationships among variables. It uses the ranked distances, so tending to linearise the relationships between variables[1]. PC-ORD software version 6.08 (MJM software Design, Gleneden Beach, OR) was used. A main matrix consisting of an anonymised farm ID designation and the variables listed in Table A1 (Supplementary Methods S1) was created. The final matrix consisted of six variables and 5000 farms. An additional second matrix was created with farm ID and the probability of an epidemic occurring and the maximum size of the epidemic.

NMS was used with a Euclidian distance measure after relativizing by standard deviates of the columns. The dimensionality of the data set was first determined by plotting a measure of fit ("stress") to the number of dimensions. Optimal dimensionality was based on the number of dimensions with the lowest stress (i.e. the smallest departure from monotonicity in the relationship between distance in

the original space and resistance in the reduced ordination space). A two-dimensional solution was requested of the NMS, since the inclusion of additional dimensions did not statistically improve the fit. Two hundred and fifty iterations were used for each NMS run, using random starting coordinates. Several NMS runs were performed for each analysis to ensure that the solution was stable and represented a configuration with the best possible fit.

Diagnostics for the NMS ordination model are presented in Table A2 (Supplementary Methods S1). The final NMS solution was two dimensional and explained 90.1% (cumulative r^2=0.901, Axis 1 r^2=0.705 and Axis 2 r^2=0.196) of the variation in the risk of CSF spread and also explained more variation than expected by chance (Monte Carlo test p=0.099). Final stress for the two-dimensional solution was 13.7 and final instability was 0 with 130 iterations. Final stress outcomes comprised between 10 and 20 were determined as showing a fair ordination for ordination solutions[1]. Number of iterations is the number of steps that NMS performed to find the final solution[1].

The results of the NMS models are shown using 2D ordination graphs of the distance between sample units which approximates dissimilarity in the estimated risk of CSF spread (Supplementary Fig. 1). The 80% confidence ellipses further added to discriminate between groups of interest using the “car” package[2] in the R statistical software version 3.1.1[3]. Variables used in the NMS analysis are shown as vectors; the direction indicates positive and negative correlation and the length along the axis (continuous variables only) depends on the strength of the correlation on that axis. The strength of the correlation along the NMS axes for continuous variables was measured using Kendall’s τ nonparametric correlation coefficient. Significance of the τ correlation was determined using tables of critical values[4]. Significance of the categorical variables were assessed using Multi-response Permutation Procedures (MRPP) analysis, a nonparametric procedure for testing the hypothesis of no difference between two or more groups[1]. A similar approach was used to evaluate the correlation of the risk of CSF spread, as assessed by the probability of epidemic and the maximum epidemic size, with the NMS axes. The strength of the correlation along the NMS axes for both the probability of epidemic take-off and the maximum epidemic size are shown in Supplementary Fig. S1, with Kendall’s correlation coefficient τ=0.270 (P<0.001) and τ=0.274 (P<0.001), respectively.

Table A2. Measures of the goodness-of-fit of the Nonmetric Multidimensional Scaling (NMS) ordination model.

Diagnostic		NMS results
Final stress[a]		13.7
Monte Carlo test[b]		0.099
Number of iterations		130
% of variation explained		
	Axis 1	70.5
	Axis 2	19.6
	Total	90.1

[a] <5: excellent, no prospect of misinterpretation; 5-10 good: no real risk of drawing false inferences; 10-20 fair: provides a useable picture; >20 poor: dangerous to interpret; 35-40 random placement of samples[1].

[b] better-than-random solution

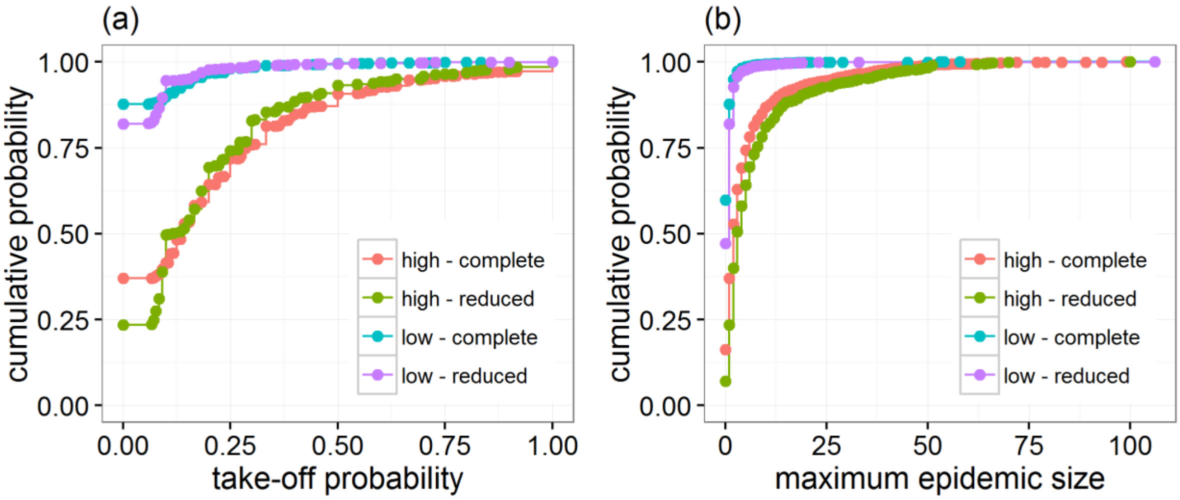

Figure A1. Difference in distribution between the whole and the reduced data set. Plots showing the empirical cumulative distribution of (a) the epidemic take-off probability, and (b) maximum epidemic size for simulations for farms located in the low and high risk areas for CSF epidemic take-off in GB in 2012. The reduced data set involved all farms that were randomly selected as the primary reported case at least ten times. Farms in high and low risk areas were defined as farms located in areas where the smoothed probability of epidemic take-off shown in Fig. 3a is <0.05 and >0.15, respectively.

Supplementary Methods S2. Details on the Bayesian inference method to estimate parameter values assessing the local between-farm spread and farm-level detection and control of CSF in Great Britain.

Data

The value of all parameters involved in both the local between-farm spread and farm-level detection and control of CSF in Great Britain were fitted using data from the CSF epidemic in East Anglia, UK that occurred in 2000 [5]. The data includes information regarding the spatial location and the time of report for all farms (*n*=16) for which CSF have been reported between August 8th to November 3rd 2000 and for which mitigation procedures were carried out. All the *n*=16 farms were used to estimate the model parameters.

Model framework and Likelihood

Here, we considered a modelling framework of the spread of the epidemic between farms similar to that used in the simulation model described in the main text. Briefly, we considered a stochastic spatio-temporal SIR model where susceptible individual farms become infectious and then removed or recovered[6]. The infectious compartment corresponds to the status where the farm is infected and is able to transmit the disease to other sites; while the recovery state means that the infection has been detected in the farm, on which mitigation procedures have been enforced within a 24-hour period post detection. In contrast with the simulation model, we assumed that (1) no movements occurred between farms during the epidemic period, and (2) the infection process would only be a function of the Euclidean distance between farms.

We further considered that epidemics occurred in a population of N farms, where the geographical location of each individual farm is known. We then assumed that epidemics start with a single initially infected farm and that an individual infected farm i would make an infectious contact with a susceptible individual j at a rate β_{ij} such as

$$\beta_{ij} = \beta_0 h_{ij} \sim K(d_{ij})$$

where β_0 is the contact rate and the function h_{ij}, together with β_0, represent the well-known spatial kernel transmission functions $K(d_{ij})$. Althought the expression of $K(d_{ij})$, and ultimately of h_{ij}, can take various forms depending on the epidemic studied and the belief of how the disease spreads, we parameterised $K(d_{ij})$ as in Boender et al.[7], such as:

$$K(d_{ij}) = \frac{k_1}{1 + \left(\frac{d_{ij}}{k_2}\right)^{k_3}},$$

where d_{ij} denotes the Euclidean distance between farms i and j $(i; j \in \{1, 2, \ldots, N\})$. Here, the distance kernel $K(d_{ij})$ allows the infection rates to decrease when the distance between two individual farms increases. Note that:

$$\beta_0 = k_1 \text{ and } h_{ij} = \frac{1}{1 + \left(\frac{d_{ij}}{k_2}\right)^{k_3}}.$$

The removal of infected farms during an epidemic mainly depends on the quality of surveillance activities. In the situation of an incursion of CSF, detection of infection is partly related to the number of animals showing clinical signs. However, the non-specific clinical signs of CSF-infected pigs, partly at early stage of the infection, may increase the difficulties to detect infected farms and create delays in the detection and reporting of disease. To account for such a delay, we assumed that an infected farm becomes detected/removed after a minimum of c ($c > 0$) days of being infectious, arbitrarily fixed to two latent periods (i.e. $2T_{\text{lat}}$=8 days) based on [8]. The infectious period of the epidemic is therefore assumed to follow a left-truncated gamma distribution:

$$R_i - I_i \sim \mathcal{TG}(\alpha, \gamma, c)$$

where I_i and R_i are respectively the infection and removal times for farm i, $\gamma \sim r_{\text{det}}$ is a constant detection rate and $\alpha \sim s_{\text{det}}$ the shape parameter of the truncated gamma distribution. The density of the truncated gamma distribution is parameterised as

$$f^{+}(R_i - I_i; \alpha, \gamma, c) = \frac{\gamma^{\alpha}}{\Gamma(\alpha, \gamma c)} (R_i - I_i)^{\alpha-1} \exp(-\gamma(R_i - I_i)) \mathbf{I}_{R_i - I_i > c}$$

where $\Gamma(\alpha, \gamma c) = \int_{\gamma c}^{\infty} \exp(-x) x^{\alpha-1} dx$ and $\mathbf{I}_{R_i - I_i > c}$ is the indicator function giving 1 if $R_i - I_i > c$ and 0 otherwise.

The likelihood of the model can therefore be expressed as:

$$L(\mathbf{R}, \mathbf{I} \mid \boldsymbol{\theta}, \gamma, \alpha) \propto \prod_{i=1, i \neq v}^{n_I} \left(\sum_{j \in y_i} \beta_{ij} \right) \times \exp(-S) \times \prod_{i=1}^{n_R} \gamma^{\alpha} \exp(-\gamma(R_i - I_i)) \frac{(R_i - I_i)^{\alpha-1}}{\Gamma(\alpha, \gamma c)}$$

where $\boldsymbol{\theta}$ is the vector of model parameters, $y_i = \{j : I_j < I_i < R_j\}$, v is the index case, and n_I and n_R are the total number of infected and removed individual farms in the population, respectively, with $n_I = n_R$ since the epidemic has ceased. We denote by S the total farm-to-farm infectious pressure during the course of the epidemic. This is the case when we consider that an infection happens only when the total pressure exerted on a susceptible by the infectious individuals is bigger than its threshold[9]. Therefore, we have

$$S = \sum_{i=1}^{n_I} \sum_{j=1}^{N} \beta_{ij} \left((R_i \wedge I_j) - (I_i \wedge I_j) \right).$$

The infection process is actually a time-dependent Poisson process and S takes into account the fact there is no event happening between times.

Bayesian inference

Data available from disease outbreaks, as it is the case here, are usually the times at which infected farms were detected as such and from which mitigation procedures have been carried out. The infection times are regularly unknown unless some diagnostic tests are available leading to some knowledge of when the infections might have occurred. But in general, no information is available on the infection times. The infection times for all infected premises during the CSF epidemic in 2000 in East Anglia would need to be inferred together with the set of model parameters $\boldsymbol{\theta}$ using data augmentation techniques. The Bayesian framework was then adopted as it provides natural approach for handling missing data problems along with the computational tool Markov Chain Monte Carlo (MCMC) methods [10-12].

The joint posterior distribution of the model parameters given the data is can be written as

$$\pi(\boldsymbol{\theta}, x \mid \mathbf{y}) \propto \pi(\mathbf{y}, x \mid \boldsymbol{\theta})\pi(\boldsymbol{\theta}),$$

where $\pi(\boldsymbol{\theta})$ is the joint prior distribution on the model parameters and $\pi(\mathbf{y}, x \mid \boldsymbol{\theta})$ is the augmented likelihood function with $\mathbf{y}$ and x representing the observed and unobserved data respectively.

Here, we considered gamma prior on both β_0 and γ. While prior and posterior conditional distributions are conjugate for β_0, this is not the case when assuming a left-truncated gamma distribution for the infectious rate parameter γ. We therefore updated γ along with the other model parameters contained in $\boldsymbol{\theta}$ using Metropolis-Hastings algorithms[13] and following a random walk scheme. The infection times are updated using a simple non-centering scheme[14,15]. For each farm i and at each MCMC step, putative infection time I_i' was proposed on the assumption of the removal process $R_i - I_i' \sim \mathcal{TG}(\alpha, \gamma, c)$. We accept I_i' with probability

$$\frac{L(\mathbf{R}, \mathbf{I}', \boldsymbol{\theta})}{L(\mathbf{R}, \mathbf{I}, \boldsymbol{\theta})} \times \frac{f^{+}(R_i - I_i; \alpha, \gamma, c)}{f^{+}(R_i - I_i'; \alpha, \gamma, c)}$$

where $\mathbf{I}'$ is the vector of infection times with I_i replaced by I_i'.

Supplementary Tables

Supplementary Table S1. Effects of producer types, country of incursions and high risk period duration on the proportion of farm-level infection events due to animal movements in Great Britain. Regression coefficients and their standard errors were computed from a generalized linear model with binomial family and logit link function of factors influencing the odds (OR) of farms being infected from the movement of pigs (vs. local spread) are shown.

Variables	Estimate (SE)	P	OR (95% CI)	P_{LRT}	%Dev.
Intercept	-0.104 (0.012)	<0.001			
**High risk period duration**				<0.001	3%
2 weeks	-0.109 (0.014)	<0.001	0.90 (0.87 – 0.92)		
4 weeks	Ref.		1.0		
6 weeks	-0.013 (0.011)	0.270	0.99 (0.97 – 1.01)		
8 weeks	0.035 (0.011)	0.001	1.03 (1.01 – 1.06)		
**Producer types of incursion**				<0.001	78%
Assured producers	Ref.		1.00		
Non-assured producers	-0.607 (0.013)	<0.001	0.54 (0.53 – 0.56)		
Small producers	-1.500 (0.011)	<0.001	0.22 (0.22 – 0.23)		
**Country of incursion**				<0.001	19%
England/Wales	Ref.		1.00		
Scotland	1.509 (0.027)	<0.001	4.52[a] (4.29 – 4.77)		
**Country x Producer types**				0.002	<1%
Scotland x Assured producers	Ref.		1.00		
Scotland x Non-assured producers	0.127 (0.047)	0.007	1.14 (1.03 – 1.25)		
Scotland x Small producers	-0.054 (0.044)	0.215	0.95 (0.87 – 1.03)		

Ref.: reference category; P: Ward's P-value; P_{LRT}: likelihood ratio test statistics P-value; CI: confidence interval; %Dev: percent of the null deviance explained.

Pseudo-R^2 = 0.99

[a] Interpretation: the odds of pig producers to be infected from the movement of pigs in epidemics generated from incursions in assured commercial farms from Scotland was 4.52 (95% CI 4.29 – 4.77) times higher than from incursions in assured commercial farms from England/Wales.

Supplementary Table S2. Comparison of variable influence on the risk of CSF spread in GB between epidemics generated by a single incursions event in low and high risk areas.

	Low risk areas			High risk areas		
**Parameters**	$\boldsymbol{D_{Tk}}$	$\boldsymbol{D_k/D_{Tk}}$	**Rank**	$\boldsymbol{D_{Tk}}$	$\boldsymbol{D_k/D_{Tk}}$	**Rank**
**Probability of epidemic take-off**						
R_0	0.014	7%	6	0.032	3%	5
T_{inf}	0.079	68%	4	0.065	31%	4
T_{lat}	0.018	17%	5	0.026	8%	6
k_1	0.396	78%	2	0.272	72%	2
k_2	0.675	87%	1	0.626	86%	1
k_3	0.135	69%	3	0.121	42%	3
r_{det}	0.005	<0.1%	9	0.018	6%	8
s_{det}	0.006	<0.1%	7	0.02	<0.1%	7
c_M	0.006	<0.1%	7	0.018	<0.1%	8
**Maximum epidemic size**						
R_0	0.018	6%	8	0.021	5%	7
T_{inf}	0.047	38%	6	0.044	41%	5
T_{lat}	0.019	21%	7	0.016	19%	8
k_1	0.451	66%	2	0.417	72%	2
k_2	0.634	70%	1	0.615	76%	1
k_3	0.186	49%	3	0.145	57%	3
r_{det}	0.052	37%	4	0.047	45%	4
s_{det}	0.049	49%	5	0.041	51%	6
c_M	0.006	<0.1%	9	0.002	<0.1%	9
**Median epidemic size**						
R_0	0.063	2%	8	0.043	21%	7
T_{inf}	0.401	13%	3	0.054	37%	6
T_{lat}	0.142	15%	7	0.027	15%	8
k_1	0.595	25%	2	0.402	58%	2
k_2	0.745	33%	1	0.573	63%	1
k_3	0.327	22%	4	0.175	55%	3
r_{det}	0.302	24%	6	0.123	46%	5
s_{det}	0.319	17%	5	0.154	51%	4
c_M	0.042	5%	9	0.006	<0.1%	9
**Proportion of infection due to animal movements**						
R_0	0.172	1%	6	0.09	13%	6
T_{inf}	0.115	19%	8	0.111	14%	4
T_{lat}	0.034	12%	9	0.092	10%	5
k_1	0.136	35%	7	0.432	64%	2
k_2	0.548	49%	1	0.854	78%	1
k_3	0.416	13%	2	0.264	33%	3
r_{det}	0.232	7%	5	0.041	7%	8
s_{det}	0.358	8%	3	0.043	19%	7
c_M	0.294	9%	4	0.036	8%	9

Variable's influence was measured using global sensitivity analysis.

D_k and D_{Tk} correspond to the first-order (direct) and total sensitivity indices for the *k*th variable tested in the global sensitivity analysis, respectively.

Supplementary Figures

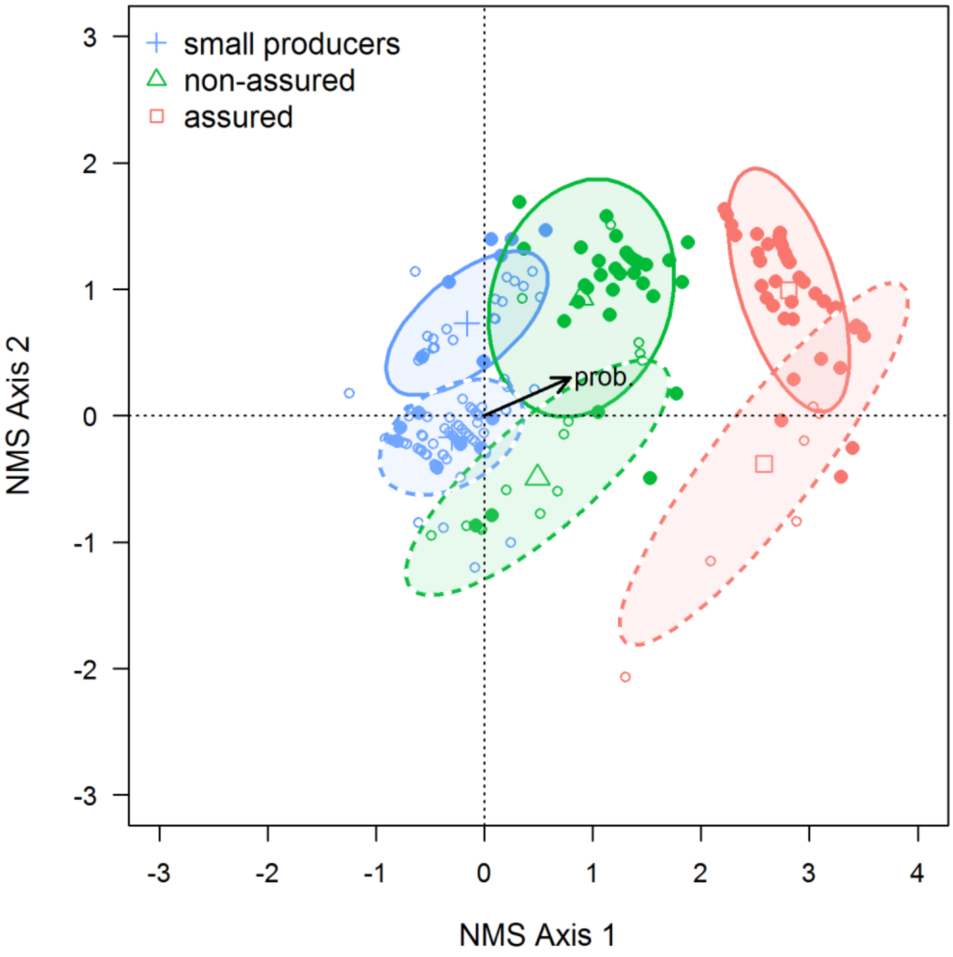

Supplementary Figure S1. Nonmetric mulitdimensional scaling (NMS) ordination of the simulation of CSF spread. The nonmetric multidimensional scaling (NMS) final solution was two dimensional and explained 90.1% of the variation in the risk of CSF spread. NMS Axis 1 shows the influence of the producer type of the index case on the risk of CSF spread, whereas NMS Axis 2 shows the influence of showing records of moving at least one pig to another producer. Solid dots represent the NMS location of the 104 producers with a probability of epidemic take-off >0.5. Small circles represent the NM location of 100 randomly-selected producers with a probability of epidemic take-off ≤0.5. Crosses, triangles and squares represent the geometric center (or centroid) of the NMS location of farms (whether they moved pigs or not) described as small producers, non-assured commercial producers and assured commercial producers, respectively. Ovals indicate the 80% confidence ellipses around the centroid for the different producer types which sent (solid) or not (dashed) pigs to at least another producer. The correlation of the risk of CSF spread, as assessed by the probability of epidemic (prob.), with the NMS axes are shown as vectors radiating from the centroid of points; the direction indicates whether the correlation with each NMS axis is positive or negative but does not indicate the strength of the relationship.

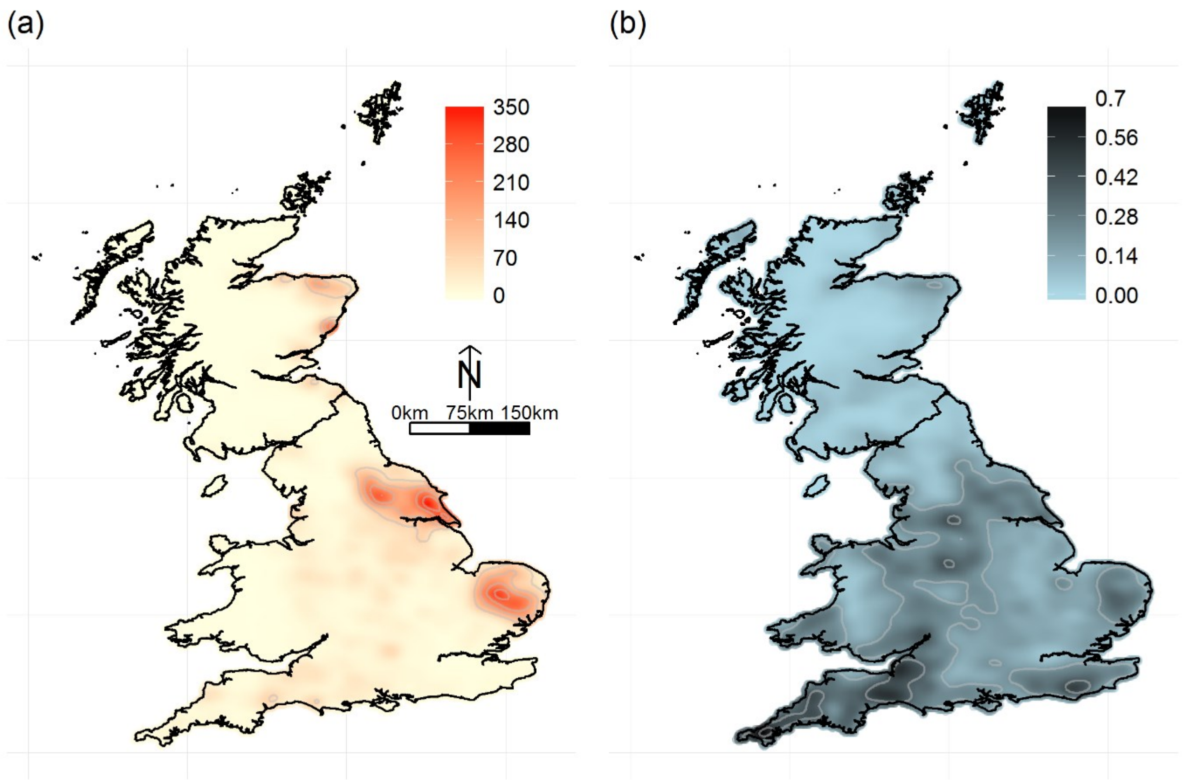

Supplementary Figure S2. Spatial distribution of pigs and pig farms in Great Britain. Maps showing the smoothed number of (a) pigs and (b) pig farms per squared kilometers. Smoothed estimates of density values were computed using the kernel intensity ratio method[16] and using a fixed 10km bandwidth. Maps were created in R version 3.2.5[3,17].

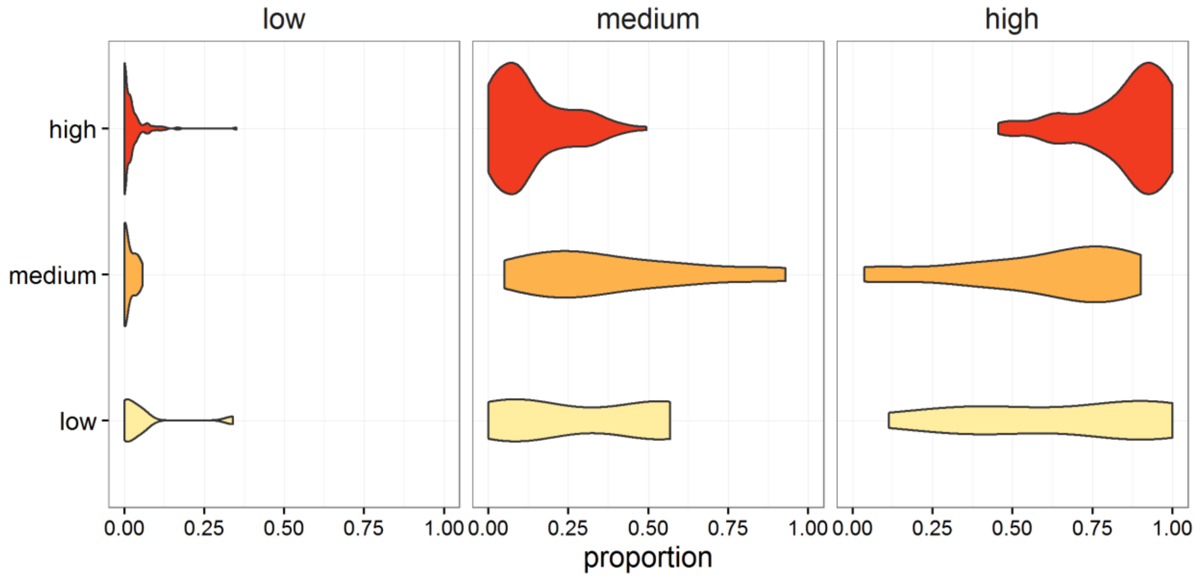

Supplement Figure S3. Proportion of producers located in a low, medium or high risk areas involved in epidemics of >50 infected premises generated by incursions in each risk areas. Here, rows indicate the risk area of the primary case (incursion), whereas columns indicate the risk area of the producers that are involved in each epidemics. Low, medium and high risk areas are defined as are areas in Fig. 3a where the smoothed probability of epidemic take-off is ≤0.05, between 0.05 and 0.15, and >0.15, respectively. The thickness of the shapes is proportional to the density of data points along the x-axis.

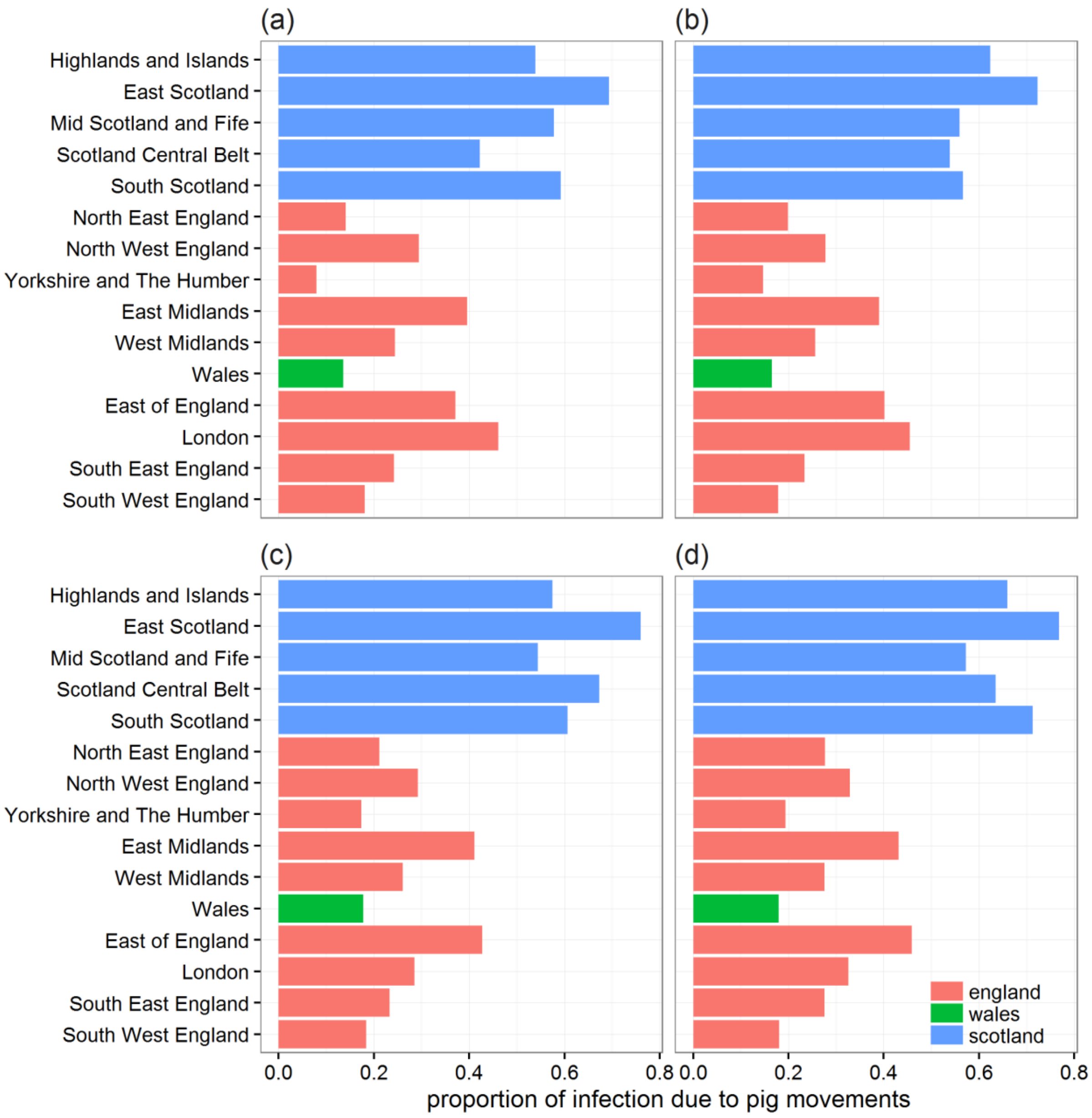

Supplementary Figure S4. Changes in the proportion of CSF infection due to pig movements from epidemics occurring throughout Great Britain. Here is shown the proportion of infections due to pig movements (vs. due to local spread) for epidemics generated from single incursions in each considered region of GB when the high risk period lasted for (a) 2 weeks, (b) 4 week, (c) 6 weeks and (d) 8 weeks. Boundaries of each considered regions are displayed in Fig. 5 and are ordered in order of latitude (from the Southernmost to the Northernmost).

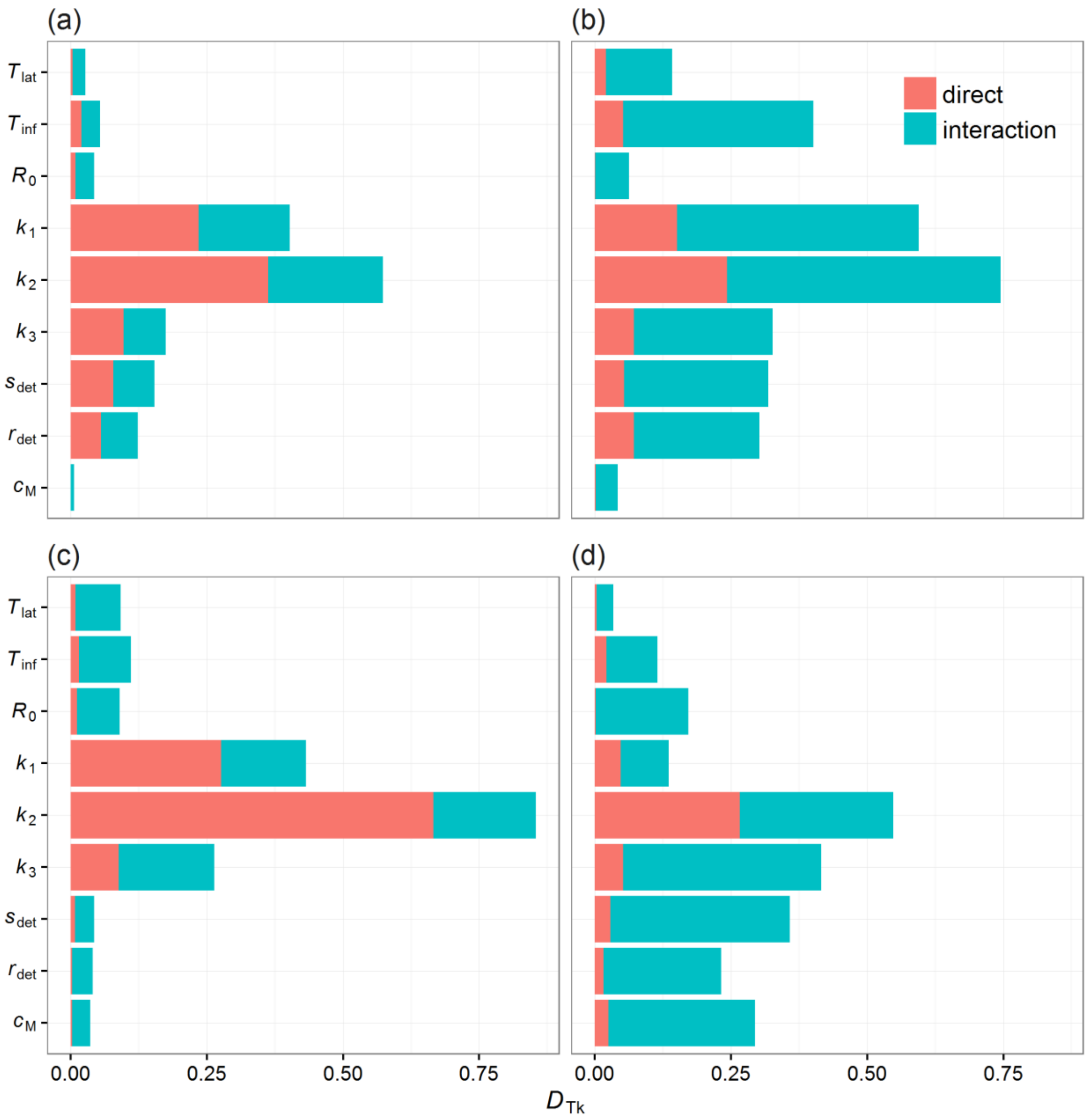

Supplementary Figure S5. Influence of the model's parameters. Results of the global sensitivity analysis on (a,b) the median epidemic size for single incursion events occurring in either (a) high or (b) low risk areas; and (c,d) on the proportion of infection events due to animal movement for epidemics generated from single incursion events occurring in either (c) high or (d) low risk areas. Influence of the model's parameters was measured by the total sensitivity index (D_{Tk}), which captures the overall effect of parameter variations, including direct effects and interactions between model parameters. Parameters definition and range of values considered in the analysis are given in Table 2. Low and high risk areas are defined as are areas in Fig. 3a where the smoothed probability of epidemic take-off is ≤0.05 and >0.15, respectively.

References

1 McCune, B., Grace, J. B. & Urban, D. L. *Analysis of Ecological Communities*. 304 (MjM Software Design, 2002).

2 Fox, J. & Weisberg, S. *An R Companion to Applied Regression*. Second Edition edn, (Sage, 2011).

3 R Development Core Team. *R: A Language and Environment for Statistical Computing*. https://cran.r-project.org/ (R Foundation for Statistical Computing, 2016).

4 Sokal, R. R. & Rohlf, F. J. *Biometry: the principles and practice of statistics in biological research*. Fourth edn, 937 (W. H. Freeman and Co., 2012).

5 Paton, D. in *Trends in Emerging Viral Infections of Swine* (eds Antonio Morilla, Kyoung-Jin Yoon, & Jeffrey J. Zimmerman) 153-158 (Iowa State Press, 2002).

6 Dawson, P. M., Werkman, M., Brooks-Pollock, E. & Tildesley, M. J. Epidemic predictions in an imperfect world: modelling disease spread with partial data. *Proceedings of the Royal Society of London B: Biological Sciences* **282**, doi:10.1098/rspb.2015.0205 (2015).

7 Boender, G. J. *et al.* Risk Maps for the Spread of Highly Pathogenic Avian Influenza in Poultry. *PLoS Comput Biol* **3**, e71, doi:10.1371/journal.pcbi.0030071 (2007).

8 Backer, J. A., Hagenaars, T. J., van Roermund, H. J. W. & de Jong, M. C. M. Modelling the effectiveness and risks of vaccination strategies to control classical swine fever epidemics. *J R Soc Interface* **6**, 849-861, doi:10.1098/rsif.2008.0408 (2009).

9 Sellke, T. On the Asymptotic Distribution of the Size of a Stochastic Epidemic. *Journal of Applied Probability* **20**, 390-394, doi:10.2307/3213811 (1983).

10 O'Neill, P. D. & Roberts, G. O. Bayesian inference for partially observed stochastic epidemics. *Journal of the Royal Statistical Society: Series A (Statistics in Society)* **162**, 121-129, doi:10.1111/1467-985X.00125 (1999).

11 Jewell, C. P., Keeling, M. J. & Roberts, G. O. Predicting undetected infections during the 2007 foot-and-mouth disease outbreak. *Journal of the Royal Society Interface* **6**, 1145-1151, doi:10.1098/rsif.2008.0433 (2009).

12 Gamado, K. M., Streftaris, G. & Zachary, S. Modelling under-reporting in epidemics. *J. Math. Biol.* **69**, 737-765, doi:10.1007/s00285-013-0717-z (2013).

13 Metropolis, N., Rosenbluth, A. W., Rosenbluth, M. N., Teller, A. H. & Teller, E. Equation of State Calculations by Fast Computing Machines. *The Journal of Chemical Physics* **21**, 1087-1092, doi:doi:http://dx.doi.org/10.1063/1.1699114 (1953).

14 Neal, P. & Roberts, G. A case study in non-centering for data augmentation: Stochastic epidemics. *Statistics and Computing* **15**, 315-327, doi:10.1007/s11222-005-4074-7.

15 Papaspiliopoulos, O., Roberts, G. O. & Skold, M. A General Framework for the Parametrization of Hierarchical Models. 59-73, doi:10.1214/088342307000000014 (2007).

16 Bithell, J. F. An application of density estimation to geographical epidemiology. *Statistics in Medicine* **9**, 691-701 (1990).

17 Bivand, R. & Lewin-Koh, N. maptools: Tools for reading and handling spatial objects. R package version 0.8-39. (2016). <http://CRAN.R-project.org/package=maptools>.
